# Biosocial Determinants of Persistent Schistosomiasis among Schoolchildren in Tanzania despite Repeated Treatment

**DOI:** 10.3390/tropicalmed2040061

**Published:** 2017-12-04

**Authors:** Rose E. Donohue, Kijakazi O. Mashoto, Godfrey M. Mubyazi, Shirin Madon, Mwele N. Malecela, Edwin Michael

**Affiliations:** 1 Department of Biological Sciences, University of Notre Dame, Notre Dame, IN 46556, USA; rdonohue@nd.edu; 2 National Institute for Medical Research, P.O. Box 9653, 3 Barack Obama Drive, 11101 Dar es Salaam, Tanzania; kmashoto@nimr.or.tz (K.O.M.); gmmubyazi@gmail.com (G.M.M.); 3 Department of International Development, London School of Economics & Political Science, Houghton Street, London WC2A 2AE, UK; s.madon@lse.ac.uk; 4 Department of Management, London School of Economics & Political Science, Houghton Street, London WC2A 2AE, UK; 5 Tanzania Commission for Science and Technology (COSTECH), P.O. Box 4302, Ali Hassan Mwinyi Road, Kijitonyama, 14113 Dar es Salaam, Tanzania; mwelentuli@gmail.com

**Keywords:** schistosomiasis, social determinants, integrated control, persistence, Tanzania, water, sanitation and hygiene (WASH)

## Abstract

Schistosomiasis is a parasitic disease endemic to Tanzania and other countries of the global south, which is currently being addressed through preventive chemotherapy campaigns. However, there is growing recognition that chemotherapy strategies will need to be supplemented to sustainably control and eventually eliminate the disease. There remains a need to understand the factors contributing to continued transmission in order to ensure the effective configuration and implementation of supplemented programs. We conducted a cross-sectional questionnaire, to evaluate the biosocial determinants facilitating the persistence of schistosomiasis, among 1704 Tanzanian schoolchildren residing in two districts undergoing a preventive chemotherapeutic program: Rufiji and Mkuranga. A meta-analysis was carried out to select the diagnostic questions that provided a likelihood for predicting infection status. We found that self-reported schistosomiasis continues to persist among the schoolchildren, despite multiple rounds of drug administration.Using mixed effects logistic regression modeling, we found biosocial factors, including gender, socio-economic status, and water, sanitation, and hygiene (WASH)-related variables, were associated with this continued schistosomiasis presence. These findings highlight the significant role that social factors may play in the persistence of disease transmission despite multiple treatments, and support the need not only for including integrated technical measures, such as WASH, but also addressing issues of poverty and gender when designing effective and sustainable schistosomiasis control programs.

## 1. Introduction

Human schistosomiasis is a tropical parasitic disease, caused by blood-dwelling flukes, that poses a grave global health problem—at least 230 million people are thought to be infected [1], with an additional 500 million at risk of infection [2]. Considered a disease of poverty, schistosomiasis disproportionately affects the world’s least affluent. More than 90% of cases are estimated to occur in sub-Saharan Africa, with the United Republic of Tanzania harboring the second highest burden behind Nigeria [3]; prevalence estimates across Tanzania range from 12.7% to 87.6% [4]. The main agents of schistosomiasis in Tanzania are *Schistosoma haematobium* and *Schistosoma mansoni* [3], which cause urogenital and intestinal schistosomiasis, respectively. School-aged children are particularly vulnerable, as age profiles have shown that prevalence and intensity generally peak among early adolescents, particularly those aged 10–14 years, followed by decreasing prevalence and intensity through adulthood; schistosomiasis-attributed morbidities include anemia and malnutrition, both of which lead to impaired childhood development [1,5,6].

Current control efforts focus predominantly on the distribution of the anti-schistosomal drug, praziquantel, to decrease morbidity from infection. The World Health Organization (WHO) recommends targeted mass drug administration (MDA) to school-aged children (SAC) at scheduled intervals based on a community’s risk status, which is assessed by either traditional parasitological methods or a validated questionnaire method [7]. Current WHO recommendations for praziquantel treatment frequency distinguish between low-, moderate-, and high-risk communities, using pre-defined prevalence cut-off values, proposing biennial treatment for moderate-risk communities and annual treatment for high-risk communities [8]. Praziquantel is considered very efficacious; four weeks post-treatment, the drug has been found to cure 60–90% of cases and cause an 80–95% average reduction in eggs excreted from still-infected patients [9]. High safety, ease of distribution to schoolchildren, and affordability, due to drug donation, all contribute to the drug being the intervention of choice [9].

While praziquantel MDA has many recognized benefits and is the mainstay of control programs globally, there is rising concern regarding the sustainability of this approach [10,11,12,13,14,15]. Schistosomiasis transmission is maintained both by the release of eggs through either urination (*S. haematobium*) or defecation (*S. mansoni*) into freshwater bodies inhabited by snail intermediate hosts, and by human contact with water sources harboring the infectious form of the parasite, cercariae. Because praziquantel does not address the underlying determinants of disease and is not a preventive drug, reinfection can occur rapidly if individuals are re-exposed to cercariae-infested water sources [16]. For example, studies evaluating the effects of exclusive MDA control programs have found that while schistosomiasis prevalence may dramatically drop during the control programs, marked rebounding of infection to near or exceeding the baseline prevalence can occur within years of MDA cessation [17,18].

Given the challenges posed by opting for exclusive MDA, policymakers seeking sustainable control and eventual parasite elimination are beginning to focus on incorporating additional efforts beyond traditional targeted preventive chemotherapy. One major evolving effort is concentrated on stopping the human activities that allow the transmission cycle to persist. This has led to calls for the integration of strategies, such as water, sanitation, and hygiene (WASH)-related efforts, with drug treatment as a more effective socioecologically-informed measure for achieving the sustainable control of schistosomiasis, and indeed water-related infectious diseases in general [10,13,19,20,21].

Despite the potential of such integrated approaches, there is also increasing recognition that a major need for the effective configuration and implementation of these measures in the field is gaining an improved understanding of the ecological, behavioral, and social risk factors that together contribute to transmission of infection among different focal groups in endemic communities [22,23,24,25]. The identification of such factors, particularly those social variables that underlie persistent transmission despite repeated MDAs of communities, will be crucial to ensuring that the supplemented control program effectively disrupts the trenchant bio-social processes that allow continued pathogen transmission in endemic communities [22,23,24,25]. Recent work on challenges faced during the endgame phase of parasite control has highlighted this important need to better understand the social factors that can govern the changed epidemiology of transmission as operational targets near [26].

Here, our primary aim is thus to undertake a bio-social study of the determinants that facilitate the persistence of schistosomiasis infection among schoolchildren residing in an endemic area undergoing an active MDA program. In this paper, we define persistent schistosomiasis as the continued transmission of schistosomiasis at the community-level for any reason (e.g., residual infection, re-infection) despite multiple rounds of MDA. We developed and implemented a questionnaire-based field investigation to achieve two major aims: (1) assessment of schistosomiasis infection at the individual level; and (2) identification of the bio-social determinants that allow infection to persist among schoolchildren. This study was conducted during 2015 in Pwani Region, Tanzania, where praziquantel MDA has been administered to schoolchildren since 2011.

## 2. Materials and Methods

### 2.1. Overview of Study

**Figure 1:**
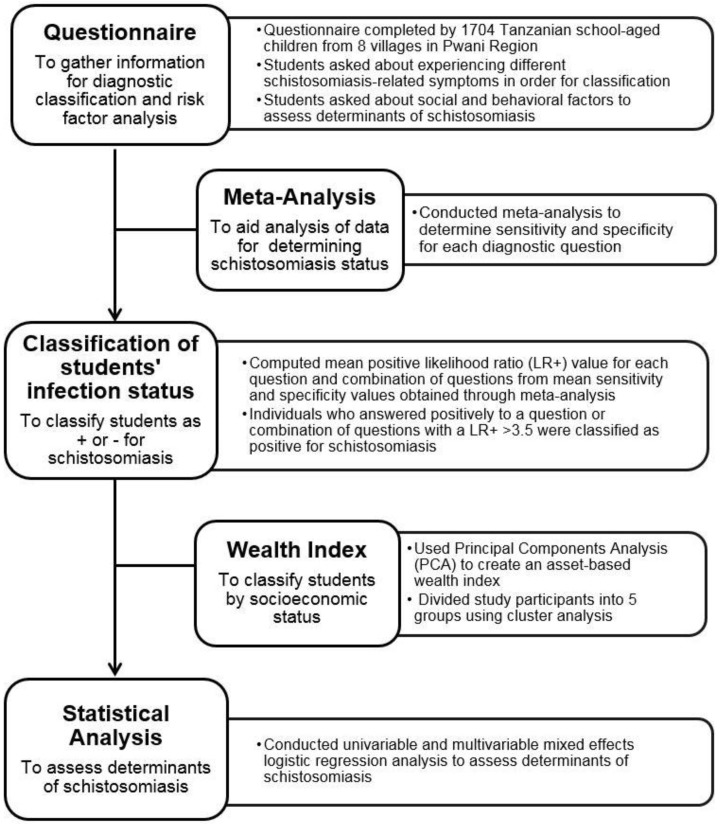
Overview of study framework.

The workflow for carrying out this study is depicted schematically in Figure 1, and comprised the following steps. First, we conducted a cross-sectional questionnaire of schoolchildren in two neighboring schistosomiasis-endemic districts in Tanzania. To guide the assessment of schistosomiasis infection status using this questionnaire, we conducted a meta-analysis to identify the morbidity-related questions that indicated a high likelihood of schistosomiasis infection. The results from this analysis were subsequently used to classify the students’ infection status in this study. Similarly, to obtain a variable representing socio-economic status, we constructed a wealth index based on the household assets assessed in the questionnaire. Finally, we used the constructed wealth index variable, schistosomiasis status, and other socio-biological variables assessed in the questionnaire to identify factors facilitating persistent schistosomiasis using a mixed effects logistic regression analysis (Figure 1).

### 2.2. Questionnaire

A cross-sectional individual-based questionnaire was conducted in 2015 among schoolchildren between the ages of 10 and 16 in two districts of Pwani (Coast) Region, Tanzania. Schoolchildren from four villages in both Rufiji and Mkuranga districts were selected to participate in this questionnaire. The questionnaire consisted of three components: (1) a morbidity-related questionnaire to derive schistosomiasis infection status, (2) a household asset-based questionnaire to classify children into socioeconomic classes, and (3) a risk assessment questionnaire to capture determinants that may underlie schistosomiasis infection among schoolchildren. Field researchers at Tanzania’s National Institute for Medical Research conducted the questionnaire, entered the data, and securely coded the data for the protection of participants.

#### 2.2.1. Study Area

Pwani Region is a coastal region in eastern Tanzania that borders the Indian Ocean and surrounds the city and region of Dar es Salaam. Mkuranga and Rufiji districts are neighboring districts within Pwani Region that were similarly classified as moderate-risk communities for *S. haematobium* in 2004 using the World Health Organization’s blood-in-urine questionnaire method; 9.3% and 9.7% of schoolchildren were classified as positive by this questionnaire method in Rufiji and Mkuranga, respectively [4]. A 2014 parasitological study confirmed that Mkuranga district remains schistosomiasis-endemic, finding a 9.2% *S. haematobium* prevalence by microscopic examination of urine among 420 school-aged children [27]. *S. mansoni* is also thought to be prevalent at lower levels in this region (MM, personal communication). MDA for praziquantel was initiated in both Rufiji and Mkuranga districts during 2011. Praziquantel was re-administered to schoolchildren in Rufiji in 2012 and 2014, while praziquantel was re-administered to Mkuranga schoolchildren in 2013.

#### 2.2.2. Study Variables

Infection status (positive/negative) for schistosomiasis served as the dependent variable. Due to the similar risk factors and transmission cycle, individuals classified as positive for *S. haematobium* and/or *S. mansoni* by the questionnaire method were grouped together and considered schistosomiasis-positive.

Independent variables analyzed included age, sex, parents’ occupations, highest level of education obtained by parents, latrine usage, safety of the main source of drinking water, wealth index, and frequency of contact with rivers, dams, lakes, and springs. The variable, ‘main source of drinking water’, was dichotomized into the categories ‘safe’ and ‘unsafe’, based on the suitability of the water source for accommodating the intermediate snail hosts [20]. Safe water sources included wells and rainwater, while unsafe water sources included freshwater bodies, such as dams, lakes, and rivers. Latrine usage was also dichotomized, separately grouping children who reported using a latrine at home or at their neighbors’ homes and children who did not use latrines.

### 2.3. Meta-Analysis

In this study, we sought to classify individuals as schistosomiasis-positive or negative using a morbidity-related questionnaire. While questionnaires have been used and validated to classify the risk level of communities for *S. haematobium* [28], their ability to correctly classify individuals has garnered less work. Community-level validation of questions has normally been conducted by (1) determining the pooled sum of reported positive questionnaires and positive parasitological tests in the same population, (2) classifying high risk communities, using a pre-defined threshold for each method, and (3) using diagnostic measures such as the positive and negative predictive values to evaluate the questionnaire’s ability to appropriately classify schools [28,29]. Alternatively, individual-level analysis compares each individual’s answer on the questionnaire to each individual’s corresponding parasitological test result. While Lengeler et al. [29] conducted a review that focused on questionnaire use at the community level, suggestions were made for questions to be asked at the individual level. We sought to determine how well these particular morbidity-related questions could correctly diagnose an individual’s schistosomiasis status. We did this primarily by conducting a meta-analysis of the sensitivity and specificity of several morbidity-related questions for correctly classifying a schistosomiasis infection in an individual.

#### 2.3.1. Deriving Questions to Classify Infected Individuals

Three questions were selected based on the Lengeler et al. review to determine *S. haematobium* infection status [29]. Schoolchildren were asked if they had noted blood in their urine in the past two weeks, if they had experienced any pain while urinating in the past two weeks, and if they had a previous history of schistosomiasis. For *S. mansoni*, four questions were asked to determine infection status. Schoolchildren were asked if they had noted the presence of blood in the stool in the past two weeks, experienced bloody diarrhea in the past two weeks, experienced lower abdominal pain in the past two weeks, and if they had a history of schistosomiasis infection. In order to systematically explore the reliability of these questions, a meta-analysis was conducted, to determine the sensitivity and specificity of each question for correctly classifying individuals, for both *S. mansoni* and *S. haematobium*.

#### 2.3.2. Meta-Analysis Procedure

We followed standard meta-analysis procedures, in accordance with the PRISMA guidelines [30] for summarizing diagnostic tool performance, to determine the reliability of questions used to determine schistosomiasis infection, as follows.

#### 2.3.3. Study Identification

Studies published prior to 1 October 2015 that met the inclusion criteria were identified using the publication database, PubMed, and by scanning references of relevant retrieved articles, using the search strategy (schisto* OR mansoni OR bilhar* OR haematob*) AND (questionnaire* OR diagnostic* OR assessment* OR survey* OR sensitivit* OR specificit* OR hematuria OR ‘blood in stool’ OR ‘diarrhea’ OR ‘history of schistosomiasis’ OR dysuria). Studies were first screened by title for relevance, then screened by abstract, and studies deemed to be relevant after these two screenings were included in the full-text assessment for eligibility.

#### 2.3.4. Study Inclusion Criteria

Studies were included if they met the following eligibility criteria: (1) study examined diagnostic question and *S. haematobium* or *S. mansoni* infection outcome; (2) a 2 × 2 table was reported or could be reconstructed for each question and schistosomiasis outcome; (3) *S. mansoni* and *S. haematobium* outcomes were assessed using gold standard tests for parasitological examinations (microscopic examination of excreta) [31]; (4) individuals sampled were a representative sample from the community (i.e., studies using only hospital patients excluded); (5) both genders were included in study; and (6) article was written in English. Studies meeting all the eligibility criteria were included in the meta-analysis.

#### 2.3.5. Data Extraction

The following information was extracted, for each diagnostic question, from the selected studies: name of the first author, year of publication, country, sample size, prevalence, ages of study population, and data for 2 × 2 contingency tables, consisting of true positives (TP), false positives (FP), false negatives (FN), and true negatives (TN).

#### 2.3.6. Quality Assessment

To evaluate the quality of included studies, we assessed studies at the outcome level using the QUADAS-2 tool, which assesses the risk of bias and applicability across four domains: patient selection, index test, reference standard, and flow and timing [32].

#### 2.3.7. Analysis Procedure

While pooling sensitivity and specificity separately has been criticised for its inability to account for potential negative correlations existing between the diagnostic measures, two statistically superior methods have emerged in the literature: hierarchical summary receiving operating characteristic model (HSROC) [33] and a bivariate approach [34]. Harbord et al. demonstrated that the bivariate approach and the HSROC are equivalent in most situations [35]. In this analysis, the bivariate approach was used, whereby log-transformed sensitivity and specificity are combined into a single bivariate regression model. By analyzing the two diagnostic measures jointly, the correlation between sensitivity and specificity is explicitly considered. Publication bias was assessed using Deeks funnel plot asymmetry test [36]. The meta-analysis was conducted in R using the ‘mada’ software package [37].

### 2.4. Classifying Infection Status of Individuals

While the meta-analysis produced the sensitivity and specificity of individual questions for correctly classifying schistosomiasis infection, given that multiple individual questions were asked for each disease, we also sought to determine the sensitivity and specificity of answering positively to combinations of questions. The sensitivity and specificity for combined questions were obtained by evaluating the test questions in series, meaning an individual would have to answer positively to each question to be deemed positive for that group of test questions. Sensitivities for questions asked in series were multiplied together: $se_{q1\;and\;q2}=se_{q1}\times se_{q2}$. Specificities for questions asked in series were computed using the following formula: $sp_{q1\;and\;q2}=1-(1-sp_{q1})\times \left(1-sp_{q2}\right)$.

After we computed the sensitivity and specificity values for individual questions from the meta-analysis and the sensitivity and specificity values for combinations of questions utilizing the equations for questions asked in series, we computed one final value for each question and combination of questions that combined the sensitivity and specificity values: the positive likelihood ratio (LR+). We calculated the LR+ for each question and combination of questions, using the formula $\mathrm{LR}+=\frac{sensitivity}{1-specificity}$. The LR+ provides an estimate of how many times more likely individuals with the target disease are to test positive than individuals without the target disease [38]. The benefits of using likelihood ratios are that they can be used at the individual level and that they do not vary among different populations, because they are a ratio of sensitivity and specificity [39].

For this paper, the criteria of LR+ >3.5 was used as the cut-off, with tests exceeding an LR+ of 3.5 being considered in classifying infection status. Individuals positive for at least one of the tests with an LR+ >3.5 were classified as ‘infected’ for the purposes of the analysis. Individuals found negative for all the tests with an LR+ >3.5 were classified as ‘uninfected’. In the literature, LR+ values >2 have been used for indicating disease status, with the prognosis of disease generally increasing with LR+ values [40]. We opted to use a cut-off LR+ value of 3.5 here, to take advantage of relatively low false positive rates but capture an increasing number of true positives, as a result of the diagnostic performance of the questionnaire, as further explained in the results.

### 2.5. Wealth Index

Prior to statistically analyzing the determinants facilitating schistosomiasis persistence, we sought to classify children by relative socioeconomic status to include this variable in the analysis; in order to do this, a wealth index based on household assets was constructed using the methods developed by Filmer and Pritchett [41]. Asset-based indices better capture long-term socioeconomic status than household income, as household assets are less subject to short-term fluctuations than income, which may be particularly affected by issues such as seasonality [41]. Given that the acquisition of schistosomiasis is likely more a function of long-term socioeconomic status than immediate family wealth and as such wealth indices are also preferred as they are thought to be less subject to recall bias and issues of measurement [42], an asset-based wealth index was thus developed and utilized in this study.

The constructed wealth index included ownership of the following assets: house, latrine, land, radio, television, motorcycle, bicycle, cell phone, and refrigerator. Assets were coded as binary variables (1 = yes, 0 = no). A principal components analysis (PCA) was applied to determine relative weights of each asset contributing to a composite wealth index. Although PCA is a multivariate statistical method that can be used to reduce the number of variables into a smaller set of uncorrelated indices or components, where each component is a linear weighted combination of the initial variables [43], the first principal component is generally considered to represent economic status when applied to economic asset data [44], and was thus used to derive the wealth index in this study. After a weight was assigned to each asset variable, component scores were calculated for individuals, and a cluster analysis was used to differentiate between socio-economic groups. To find the optimal number of clusters in the partitioning of the socioeconomic scores, we compared the results of multiple indices developed for this purpose in the R package, ‘NbClust’, to determine the most frequently-proposed number of clusters for this dataset [45]. The resulting optimal number of clusters was then used to conduct k-means clustering, and individuals were grouped by socioeconomic status based on these results.

### 2.6. Statistical Analysis

After the infection status and the wealth index variable were determined, mixed effects logistic regression modeling was used to investigate the association between potential determinants and schistosomiasis infection, with the school’s village being included as a random effect variable to account for potential dependence of the data on the village due to the focal nature of schistosomiasis and potential environmental factors that were not accounted for in the questionnaire. Infection status (positive/negative) for schistosomiasis served as the dependent variable, while the risk factors obtained directly from the questionnaire and the constructed wealth index variable served as the independent variables.

The association between individual risk factors and self-reported schistosomiasis was assessed using both univariable and multivariable mixed effects logistic regression models. Variable selection for the multivariable model was performed using least absolute shrinkage and selection operator (LASSO) penalized mixed effects logistic regression in the ‘glmmLasso’ package in R [46]. LASSO regression shrinks some coefficients and sets others to zero, allowing for both improved prediction accuracy and interpretation when compared to standard variable selection techniques [47]. The estimate for lambda was selected by performing a grid search on integers, varying from 500 to 0, to find the lambda which produced the lowest Akaike information criterion (AIC) score. Variables with non-zero coefficients were subsequently entered into a nonpenalized mixed effects logistic regression model, using the ‘lme4’ package [48].

### 2.7. Ethical Approval

This study protocol was reviewed and approved by the Institutional Review Board at the University of Notre Dame and Tanzania’s National Institute for Medical Research. Informed written consent was obtained from the schoolchildren in the presence of the field researcher prior to administration of the questionnaire.

## 3. Results

### 3.1. Meta-Analysis

A total of 7398 potentially relevant articles were identified through the search, with 7348 excluded for irrelevance or failure to meet inclusion criteria (Figure S1). A total of 368 full-text articles were assessed for eligibility, of which 39 studies from 27 sources met the inclusion criteria and were used in the meta-analysis for *S. haematobium* diagnostic questions (Table S1) [5,49,50,51,52,53,54,55,56,57,58,59,60,61,62,63,64,65,66,67,68,69,70,71,72,73,74], while 36 studies from 23 sources were used in the meta-analysis for *S. mansoni* diagnostic questions (Table S2) [29,75,76,77,78,79,80,81,82,83,84,85,86,87,88,89,90,91,92,93,94,95,96]. A random effects model was used to account for between-study heterogeneity, as significant heterogeneity (*p* < 0.001) was observed for each diagnostic question’s meta-analysis, based on the chi-squared tests for equality of sensitivities and specificities. The presence of a publication bias, assessed using Deeks funnel plot asymmetry test, was observed among the data for the blood in stool question (*p* < 0.001) and the abdominal pain question (*p* = 0.053), but not among the data for the blood in urine (*p* = 0.597), history of schistosomiasis for *S. haematobium* (*p* = 0.192), pain during urination (*p* = 0.834), bloody diarrhea (*p* = 0.146), or history of schistosomiasis for *S. mansoni* (*p* = 0.493) questions (Figures S2 and S3). The quality assessment indicated none of the included studies were at high risk of bias across all four domains (Figures S4 and S5).

**Figure 2:**
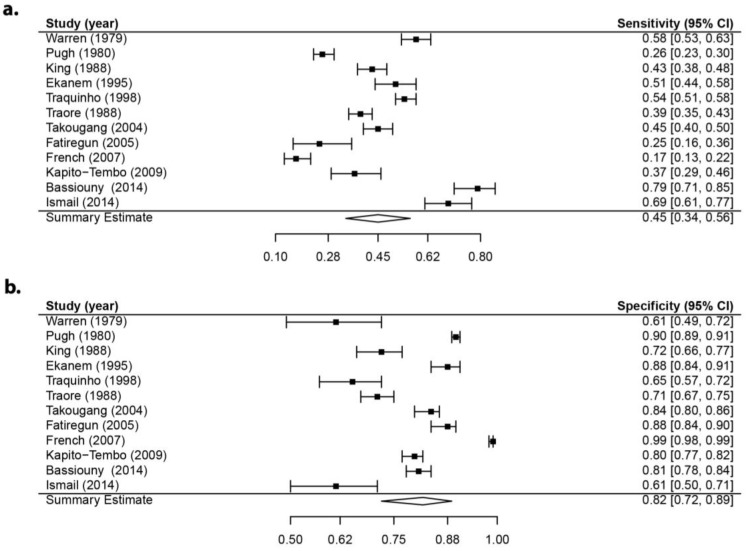

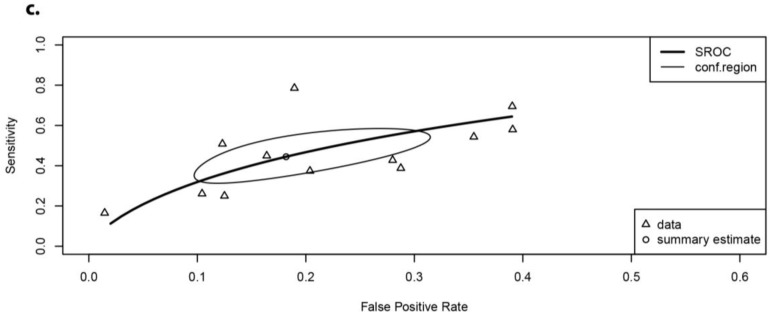
Sensitivity forest plot, specificity forest plot, and summary receiving operating characteristic (SROC) plot for pain during urination question (*S. haematobium*): (**a**) sensitivity forest plot; (**b**) specificity forest plot; and (**c**) SROC curve with summary sensitivity and false positive rate (1-specificity) (circle) and the 95% confidence region (ellipse). Each triangle represents the summary sensitivity and false positive rates from one study.

The sensitivity and specificity forest plots and the summary receiving operating characteristic (SROC) curve results for one of the diagnostic questions for *S. haematobium*—pain during urination—are presented in Figure 2 [5,49,51,55,56,58,60,61,62,67,68,70]. These results indicate that while there is heterogeneity between studies, the sensitivity and specificity obtained from the bivariate regression model for the use of this question for diagnosing *S. haematobium* in humans are estimated to be 0.45 (95% CI: 0.34–0.56) and 0.82 (95% CI: 0.72–0.89), respectively (Figure 2a,b). The corresponding SROC curve of the included studies and the summary point estimates for the pairs of sensitivity and false positive rates (1-specificity) are presented in Figure 2c. This diagnostic question did not perform ideally, which is evidenced by the curve’s presence in the upper left corner of the ROC space. Forest plots and SROC plots for all other individual questions can be found in the supplementary information (Figures S6–S11).

The diagnostic performance of individual and combined questions for *S. haematobium* and *S. mansoni* results are presented in Table 1 and Table 2, respectively. When considering the diagnostic performance of question(s) for correctly classifying individuals as schistosomiasis-positive, we generally found a low true positive rate (sensitivity), indicating the tests were missing the majority of true positives, and a low false positive rate (1-specificity), indicating the tests were yielding few false positives. While ideally one would want an increased true positive rate and decreased false positive rate, we found that, as LR+ (true positive rate/false positive rate) increased, the true positive rate and false positive rate both decreased, while, as LR+ decreased, the true positive rate and false positive rate both increased. Therefore, to take advantage of our relatively low false positive rates but capture an increasing number of true positives, we opted to use a moderately low LR+ cutoff value (3.5) in this analysis to balance obtaining adequate numbers of positive cases while keeping the false positive rate sufficiently low (Figure S12).

The best diagnostic question for *S. haematobium* was the presence of blood in urine, which returned the highest sensitivity (0.508), specificity (0.868), and positive likelihood ratio (3.85) among the three questions asked (Table 1). Presence of blood in urine was the only question to which a positive response alone resulted in positive schistosomiasis classification. Pain during urination was the second best diagnostic question, in terms of the three measures of diagnostic accuracy assessed, while history of schistosomiasis infection performed the poorest in all three diagnostic accuracy measures. Positive answers to at least two of the questions each resulted in an LR+ >3.5 and subsequent positive *S. haematobium* infection classification. Overall, *S. haematobium* diagnostic questions returning an LR+ >3.5 had sensitivities ranging from 0.083 to 0.508 and specificities ranging from 0.868 to 0.995.

The diagnostic performance of individual and combined questions for *S. mansoni* results are presented in Table 2. None of the four questions alone were deemed strong enough to classify individuals as positive for *S. mansoni* infection. Presence of blood in stool returned the highest specificity and LR+, while abdominal pain returned the highest sensitivity; however, abdominal pain also had the lowest specificity and LR+. Six combinations of questions returned an LR+ exceeding 3.5, each with low sensitivity, ranging from 0.009 to 0.082 and high specificity, ranging from 0.977 to 0.999.

One hundred and fifty-one schoolchildren were classified as positively carrying a *S. haematobium* infection, 37 schoolchildren were classified as positive for *S. mansoni*, and 168 schoolchildren were classified as having a *S. haematobium* and/or *S. mansoni* infection.

### 3.2. Wealth Index

The PCA analysis results of the variables, used to derive an asset-based wealth index for measuring the socioeconomic status of the study children, are presented in Table 3. The first principal component, which is assumed to best measure socioeconomic status [44], was found to explain 20.8% of the variation in the data. Although this value is relatively low, it does lie within the range of previously-constructed wealth indices, which have found the first component to describe between 12.7% [44] to 27% [42] of the variation in various data thought to reflect wealth status. The factor scores derived from Component 1 are presented in Table 4; these show that the highest contributing variables to the wealth index were ownership of a house, latrine, and phone with factor scores of 0.37, 0.36, and 0.27, respectively. The distribution of wealth scores is left-skewed (Figure S13) and the optimal number of clusters was identified as five. The index is internally coherent, as average asset ownership varies between the five wealth clusters and increases with increasing wealth cluster (Table S3).

### 3.3. Factors Associated with Self-Reported Schistosomiasis

#### 3.3.1. General Characteristics of Study Population

A total of 1704 schoolchildren, between the ages of 10 and 16, completed the questionnaire under the supervision of a field researcher. Of these 1704 children, 1019 (59.8%) were females and 685 (40.2%) were males. General characteristics of the study population are shown in Table 5.

#### 3.3.2. Statistical Analyses

Table 6 indicates the results from the univariable analysis, conducted to determine possible associations between risk factors and self-reported schistosomiasis. Male children were significantly more likely to be infected with schistosomiasis than females (crude odds ratio (COR) 2.49; 95% CI: 1.79–3.47). The prevalence of schistosomiasis was significantly higher among schoolchildren who did not regularly use latrines (COR 2.55; 95% CI: 1.05–6.23). Schoolchildren whose main source of drinking water was unsafe were significantly more likely to experience schistosomiasis (COR 3.05; 95% CI: 1.79–5.19). River and spring contact were found to be protective among children who visited the respective water body at least once per day: significantly for river contact (COR 0.45; 95% CI: 0.25–0.81) and marginally significant for spring contact (COR 0.52; 95% CI: 0.25–1.06). No significant association existed between the frequency of lake and dam contact with schistosomiasis.

The final multivariable model for determinants of schistosomiasis-related morbidity in Tanzanian schoolchildren is presented in Table 7. Seven variables were retained by the LASSO regression: sex, latrine usage, main source of drinking water, wealth index, frequency of lake contact, frequency of river contact, and education of parent. Male children were significantly more likely to be infected with schistosomiasis than female children (AOR 2.69; 95% CI: 1.90–3.80). Children who did not regularly use latrines had marginally significant higher odds of schistosomiasis (AOR 2.33; 95% CI: 0.90–6.01). Children who used an unsafe water source for their drinking water had significantly higher odds of schistosomiasis infection (AOR 3.57; 95% CI: 2.03–6.27). Wealth index was also retained in the final model; children belonging to the upper four clusters were less likely to harbor a schistosomiasis infection than those in the poorest cluster, with cluster 5 being significant (AOR 0.38; 95% CI: 0.16–0.90) and cluster 4 being marginally significant (AOR 0.43; 95% CI: 0.18–1.04). Two of the water contact frequency variables were retained in the final model: lake and river contact. An increased frequency of lake contact was found to increase the odds of schistosomiasis among those who visited 1–4 times/week compared to never (AOR 3.25; 95% CI: 1.25–8.47). An increased frequency of river contact was found to decrease the odds of schistosomiasis among those who visited either 1+/day or 1–4 times/week compared to never. While education of parent was retained in the final model, none of the levels were significantly associated with changed odds of schistosomiasis, when compared to the reference level.

## 4. Discussion

The present study found that schistosomiasis continues to occur among schoolchildren in the Rufiji and Mkuranga districts in Tanzania, despite each having undergone 2–3 rounds of praziquantel treatment since 2011 as part of an active national control program. Using a schistosomiasis classification system, created from questions whose diagnostic potential was evaluated through meta-analysis, we attempted to evaluate potential bio-social factors that may underlie this persistence of transmission in the schoolchildren of these districts. We identified the factors—sex, wealth index, utilization of a safe source of drinking water, and frequency of water contact with rivers and lakes—as factors associated with infection in this context.

To our knowledge, our meta-analysis is the first to systematically evaluate the potential validity of specific survey questions to identify individuals with a *S. haematobium* or *S. mansoni* infection. While questionnaires have been used and validated at the community level, our results suggest that the performance of individual diagnostic questions in accurately classifying host schistosomiasis infection at the individual level was poor overall, reflecting low to moderate sensitivities and moderate to high specificities in general. In evaluating potential applications of the individual level questionnaire that have been proposed or used in the literature, including targeting of drug treatment, monitoring effects of schistosomiasis control [29], and as an inexpensive and less invasive method for conducting research [97], the low sensitivities are particularly concerning as they indicate a high false negative rate. This false negative rate may be even higher, as the traditional diagnostic gold standard used in this meta-analysis—the microscopic examination of urine or feces—has been found to demonstrate varying sensitivity, depending on the prevalence and intensity of schistosomiasis as well as the number of samples tested [31]. The questionnaire’s high false negative rate would likely preclude its use for targeting treatment, for example, as a high percentage of infected children would be missed. Rather, the use of microscopic examination of excreta or tests with even better diagnostic accuracy, such as circulating cathodic antigen (CCA) tests for *S. mansoni* [98], would be better for the purposes of targeting treatment or monitoring the effects of control, due to their higher sensitivity values. While the use of the questionnaire for monitoring the effects of control or targeting of treatment may thus not be recommended, given our findings, the use of the questionnaires for other purposes—such as for researchers seeking to evaluate social and other determinants of infection—may be appropriate, given the high likelihood that those classified as positive are truly positive. Additionally, diagnostic questions asked in series resulted in increased specificity at the expense of reduced study sensitivity. However, this trade-off means that the increasing certainty that individuals classified as positive are truly positive occurs at the expense of missing increasing numbers of truly infected individuals. Therefore, the replication of the questionnaire approach must be cautiously conducted, ensuring the aims of the user match the chosen methods.

Given the findings of the meta-analysis and the subsequent classification system devised, this study established that schistosomiasis is still prevalent among schoolchildren of the two districts, despite each district having undergone 2–3 rounds of praziquantel treatment since 2011. The classification system devised in this study, which was based on the results of the meta-analysis, used the LR+ to incorporate both study sensitivity and specificity; thus, individual questions and combinations of questions indicating positive infection status were all of high specificity (all exceeding 0.86) and low to moderate sensitivity (all less than 0.51). This means that the individuals who our classification system deemed positive have a high likelihood of being disease positive; however, our classification system may miss many individuals carrying a schistosomiasis infection. Therefore, our results would suggest that the actual disease prevalence in the study populations is likely to exceed the 9.9% found in this study. While an accurate estimation of prevalence based on the classification system used cannot be made, the classification of approximately 9.9% of the schoolchildren as schistosomiasis-positive in this study nonetheless demonstrates that schistosomiasis continues to remain a problem in the study regions, compelling the need for researchers and policymakers to explore reasons for persistence and re-evaluate the current control strategy.

Our investigation of the bio-social factors underlying the infection status of schoolchildren despite repeated MDA in the study regions comprises a first attempt to study the reasons for this continued, persistent transmission. Our study found that males had significantly higher odds of schistosomiasis infection after multiple rounds of MDA; this finding could be attributed to sex-related differences in infection or reinfection post-treatment, noncompliance with treatment, or questionnaire bias. While many previous studies have found higher odds of schistosomiasis among males [99,100,101,102,103,104,105], associations between schistosomiasis and sex have generally been attributed to differences in sex-specific water contact rather than biological susceptibility [101,102,103,106]. Although studies have suggested that sex-related differences in immunological responses may be a factor in sex-related patterns of infection [107,108], a recent study controlling for cercarial exposure concluded that sex-related differences in *S. mansoni* reinfection were due to differences in cercarial exposure [109]. Another potential explanation for the sex-related differences identified in this study could be a sex-related bias in MDA compliance rates, as identified in compliance studies for lymphatic filariasis MDA [110]; however, compliance data was unavailable in this study for evaluation. Finally, an additional consideration for this study is the method of schistosomiasis classification. Studies conducted in Tanzania exploring the effects of gender on the reliability of self-reported *S. haematobium* infection have found that the self-reported questionnaire method significantly underestimated the infection prevalence in older girls [57,105]. Reasons hypothesized for this consistent underestimation included anatomical differences, making it more difficult for females to see their urine than males, lower levels of health education, increased reluctance to divulge personal information [105] and menses confounding recognition of symptoms [57]. However, these hypotheses were not further explored in our study; therefore, additional research should be done if the questionnaire method of estimation of infection status is utilized. 

Disparities in socio-economic status were associated with infection status in this study; the constructed wealth index variable indicated that when compared to schoolchildren in the poorest cluster, schoolchildren in wealthier clusters had reduced odds of schistosomiasis. Therefore, this study suggests that the poorest children in the community are more likely to be infected despite multiple rounds of MDA; this finding could be due to infection or reinfection post-treatment or failure to receive treatment due to non-compliance or absence from school. While schistosomiasis is considered a disease of poverty [1], studies have not always found an association between socio-economic status and schistosomiasis [24,62,99], with the predominant explanation being that an individual’s contact with cercariae-infested water may not be borne out of necessity, but for reasons such as recreational use and bathing [19,24]. Studies that do report an association generally attribute this finding to lower socioeconomic classes having poorer WASH, health knowledge, healthcare, and access to treatment [19,111]; one would thus expect the measure of socio-economic status to be found insignificant in a multivariable model controlling for these factors. Should wealth index remain an independent predictor, one potential explanation proposed in the literature [112] refers to the poverty-disease cycle ,whereby schistosomiasis is both a cause and an effect of poverty [113]. An additional reason for our finding could be that poorer schoolchildren may be more likely to be nonrecipients of treatment, due to noncompliance or absence. While a lack of studies exploring compliance to praziquantel among schoolchildren offered treatment in schools prevents further evaluation, one study exploring nonrecipients of community helminthic drug adminsitration found that increasing household wealth was associated with an increased probability of receiving praziquantel [114]. 

In the present study, the specific WASH factor, utilizing a safe main source of drinking water, was found to be associated with significantly reduced odds of schistosomiasis and the WASH factor, utilizing a latrine, with marginally significant reduced odds of schistosomiasis among schoolchildren after multiple rounds of MDA, supporting previous studies calling for the need to incorporate WASH efforts to achieve sustainable control and eventual elimination of schistosomiasis. A recent meta-analysis found that individuals with access to safe water had significantly lower odds of schistosomiasis (OR: 0.53; 95% CI: 0.47–0.61), and individuals with access to adequate sanitation demonstrated lower odds of infections from *S. haematobium* (OR: 0.69; 95% CI: 0.57–0.84) and *S. mansoni* (OR: 0.59; 95% CI: 0.47–0.73) [20]. Without addressing the behaviors or infrastructural deficiencies that promote human exposure to cercariae-infested water bodies and the contamination of water bodies with excreta, infection and reinfection are likely to occur. In our multivariable model, increased lake contact generally raised a child’s odds of schistosomiasis, while increased river contact decreased a child’s odds of schistosomiasis, suggesting that perhaps lakes serve as a local source of contamination. A potential explanation for the river contact finding may relate to the ecology of the intermediate snail hosts for *S. haematobium* and *S. mansoni*, both of which may be swept away at water flow velocities exceeding 0.3 m/s, or vegetation availability and water depth [115]. A study in Kenya, which monitored local water bodies, found that the local perennial river was not cercariae-contaminated, while other local freshwater bodies were contaminated [116]. While a more in-depth study may better capture the effects of water contact by considering suitability of water bodies for transmission as well as water exposure factors, including type of activity, duration of exposure, overall degree of exposure, and geographical proximity of schools and homes to potentially infective water bodies [62,102], our findings suggest that supplementing control programs with efforts to improve the availability of safe water and sanitation is important in this context. Although the challenges of WASH-based interventions for schistosomiasis control are well-recognized, including expensive infrastructure, the need for extremely high coverage due to the considerable reproductive potential of schistosome pairs [117], and the challenge of ensuring proper use, which often requires a contextual understanding of local customs, our study underlies the importance of WASH improvements as a critical component in any holistic program aiming for sustainable schistosomiasis control and elimination.

While the importance of incorporating WASH efforts into schistosomiasis control strategies is well-understood, our findings suggest that issues of poverty and gender are also important factors to consider when designing and implementing interventions. Gender and socio-economic inequalities appear to be major factors for maintaining infection across a broad range of infectious diseases [118,119,120,121]; tackling these deeper societal issues may thus ultimately require the transformation and empowerment of communities. Our results are in line with increasing calls that, in order to successfully accomplish the control or elimination of infectious diseases, such as schistosomiasis, it may be necessary to shift how we think about control from the strictly biomedical paradigm currently used to one that recognizes the social context in which these diseases exist and persist. Recent work on socio-ecological systems and biocomplexity [25,122], ecosystem approaches to health [123], and the notion of ‘invited spaces’, where mechanisms to facilitate community participation in population health decisions are purposefully created [124,125], may serve as possible paradigms and conceptual frameworks through which we can better understand disease transmission processes in societies and derive sustainable interventions that can effectively address such factors. While this study serves as a preliminary evaluation of the role that social determinants play in maintaining schistosomiasis in communities under active control programs, it is clear that there is a need for future work that brings these, as well as more anthropological-influenced perspectives, to better understand the drivers of persistent parasitic transmission.

This study has several limitations. The classification system was not ideal; our choice of an LR+ cutoff value of 3.5 influenced the proportion of individuals classified as positive (Figure S12). While we chose this value in an attempt to include questions with the potential to capture more true positives, this came at the expense of also capturing more false positives. This implies that future studies using the questionnaire method in areas of low prevalence may need to employ larger sample sizes than those investigated in this study to capture true positives for analysis. An additional limitation existed in the constructed wealth index, which exhibited clumping and truncation of socioeconomic scores. Clumping occurs when an insufficient number of assets are included in the wealth index and therefore households are clumped together in groups holding the same assets. Truncation of the wealth index distribution is observed when the wealth index lacks variables which distinguish between similar socio-economic groups [41]. Therefore, the inclusion of additional variables in the construction of future wealth indices is needed to alleviate this problem [43]. A third limitation is our classification of ‘safe’ and ‘unsafe’ water sources, which, while consistent with work in the field of schistosomiasis [20], differs from the international standard set by the UNICEF/WHO Joint Monitoring Program, which classifies ‘improved’ and ‘unimproved’ sources of drinking water [126]. Our method classifies all wells as ‘safe’ while the international standard only considers protected wells to be ‘improved’. Finally, potential explanatory variables were not included in this questionnaire, particularly history of praziquantel treatment, water contact-related variables, and health education variables. Knowledge of individual treatment history would allow us to distinguish between those infected due to reinfection and those who have not received treatment. Key missing water contact variables that would have allowed a more nuanced interpretation in this study include reason for water contact, percentage of body exposed and duration of exposure [68,97,104,127,128,129,130]. Finally, health education questions would help determine if lack of knowledge is an underlying reason for unhygienic behavioral practices.

## Figures and Tables

**Table 1:** Diagnostic performance of each question and combination of questions for correctly classifying an individual as *S. haematobium*-positive.

*S. haematobium* Diagnostic Question(s)	Sensitivity ^1^	Specificity ^1^	LR+ ^2^	Number (+) from Questionnaire Data
Blood in urine	0.508	0.868	3.85 ^3^	28
Pain during urination	0.445	0.818	2.45	142
History of schistosomiasis infection	0.365	0.807	1.89	156
Blood in urine Pain during urination	0.226	0.976	9.41 ^3^	13
Blood in urine History of schistosomiasis infection	0.185	0.975	7.28 ^3^	8
Pain during urination History of schistosomiasis infection	0.162	0.965	4.62 ^3^	83
Blood in urine Pain during urination History of schistosomiasis infection	0.083	0.995	17.80 ^3^	19

^1^ Sensitivity and specificity for individual questions computed from meta-analysis; sensitivity and specificity for combination of questions combined in series; ^2^ LR+: positive likelihood ratio; ^3^ indicates LR+ >3.5 and subsequent positive schistosomiasis classification for this study.

**Table 2:** Diagnostic performance of each question and combination of questions for correctly classifying an individual as *S. mansoni*-positive.

*S. mansoni* Diagnostic Question(s)	Sensitivity ^1^	Specificity ^1^	LR+ ^2^	Number (+) from Questionnaire Data
Blood in stool	0.249	0.904	2.69	19
Bloody diarrhea	0.283	0.849	1.87	14
Abdominal pain	0.399	0.666	1.19	332
History of schistosomiasis	0.329	0.765	1.40	157
Blood in stool Bloody diarrhea	0.070	0.986	4.86 ^3^	5
Blood in stool Abdominal pain	0.099	0.968	3.10	19
Blood in stool History of schistosomiasis	0.082	0.977	3.63 ^3^	11
Bloody diarrhea Abdominal pain	0.113	0.950	2.24	20
Bloody diarrhea History of schistosomiasis	0.093	0.965	2.62	3
Abdominal pain History of schistosomiasis	0.131	0.922	1.67	70
Blood in stool Bloody diarrhea History of schistosomiasis	0.023	0.997	6.81 ^3^	0
Blood in stool Bloody diarrhea Abdominal pain	0.028	0.995	5.81 ^3^	6
Blood in stool History of schistosomiasis Abdominal pain	0.033	0.992	4.34 ^3^	10
Bloody diarrhea History of schistosomiasis Abdominal pain	0.037	0.988	3.13	10
Blood in stool Bloody diarrhea History of schistosomiasis Abdominal pain	0.009	0.999	8.13 ^3^	5

^1^ Sensitivity and specificity for individual questions computed from meta-analysis; sensitivity and specificity for combination of questions combined in series; ^2^ LR+: positive likelihood ratio; ^3^ indicates LR+ >3.5 and subsequent positive schistosomiasis classification for this study.

**Table 3:** Variance explained by each component of the principal components analysis (PCA) of household-based assets.

	Eigenvalue	Percentage of Variance	Cumulative Percentage of Variance
**Component 1**	1.872	20.8	20.8
**Component 2**	1.574	17.5	38.3
**Component 3**	1.185	13.2	51.5
**Component 4**	0.917	10.2	61.7
**Component 5**	0.875	9.7	71.4
**Component 6**	0.835	9.3	80.7
**Component 7**	0.625	6.9	87.6
**Component 8**	0.570	6.3	93.9
**Component 9**	0.547	6.1	100.0

**Table 4:** Summary statistics and factor scores for assets included in the wealth index.

Asset	Mean	Std Dev	Factor Score
House	0.95	0.22	0.37
Latrine	0.96	0.19	0.36
Land	0.89	0.32	0.25
Radio	0.74	0.44	0.23
TV	0.16	0.37	0.02
Motorcycle	0.26	0.44	0.12
Bicycle	0.73	0.44	0.25
Phone	0.83	0.38	0.27
Fridge	0.10	0.30	0.02

**Table 5:** General characteristics of Tanzanian schoolchildren who participated in this study (*n* = 1704).

Characteristic	*n* (%)
**Sex**	
Male	685 (40.2)
Female	1019 (59.8)
**Age**	
<13 years old	779 (45.7)
≥13 years old	925 (54.3)
**District**	
Rufiji	894 (52.5)
Mkuranga	810 (47.5)
**Village**	
Bungu	268 (15.7)
izapala	160 (9.4)
MgombaKaskazini	241 (14.1)
Mgomba Kati	248 (14.6)
Misasa	197 (11.6)
Mkamba	249 (14.6)
Njopeka	204 (12.0)
Pagae	137 (8.0)
**Education of Parent**	
No formal education	350 (20.5)
Incomplete primary education	947 (55.6)
Primary education	280 (16.4)
Incomplete secondary school	93 (5.5)
Secondary education	34 (2.0)
**Occupation of Parent**	
Agriculture or livestock keeping	1260 (73.9)
All other	444 (26.1)
**Wealth Index**	
Cluster 1 (poorest)	38 (2.2)
Cluster 2	73 (4.3)
Cluster 3	302 (17.7)
Cluster 4	487 (28.6)
Cluster 5 (least poor)	804 (47.2)
**Schistosomiasis Infection Status**	
*S. haematobium*	151 (8.9)
*S. mansoni*	37 (2.2)
*S. haematobium or S. mansoni*	168 (9.9)

**Table 6:** Univariable mixed effects logistic regression analysis of risk factors associated with self-reported schistosomiasis in Tanzanian schoolchildren (village included as a random effect variable).

Variable	COR ^1^	95% CI ^2^	*p*-Value
Sex			
Male	2.49	1.79–3.47	<0.001 *
Female	Ref	--	--
**Age**			
<13 years old	1.17	0.84–1.63	0.348
≥13 years old	Ref	--	--
**Education of Parent**			
No formal education	0.57	0.22–1.43	0.228
Incomplete primary	0.40	0.17–0.98	0.044 *
Primary education	0.52	0.21–1.30	0.161
Incomplete secondary	0.95	0.34–2.61	0.916
Secondary education	Ref	--	--
**Occupation of Parent**			
Agriculture or livestock keeping	1.01	0.68–1.45	0.964
All other	Ref	--	--
**Wealth Index**			
Cluster 1 (poorest)	Ref	--	--
Cluster 2	0.59	0.20–1.72	0.336
Cluster 3	0.68	0.28–1.62	0.382
Cluster 4	0.50	0.21–1.17	0.109
Cluster 5 (least poor)	0.47	0.20–1.09	0.077
**Latrine Use**			
Yes (home or neighbors)	Ref	--	--
No	2.55	1.05–6.23	0.039 *
**Main Source of Drinking Water**			
Safe (wells and rainwater)	Ref	--	--
Unsafe (river, dam, etc.)	3.05	1.79–5.19	<0.001*
**Frequency of River Contact**			
1+/day	0.45	0.25–0.81	0.007*
1–4 times/week	0.65	0.36–1.17	0.149
Never	Ref	--	--
**Frequency of Lake Contact**			
1+/day	0.56	0.27–1.19	0.132
1–4 times/week	1.01	0.53–1.91	0.982
Never	Ref	--	--
**Frequency of Dam Contact**			
1+/day	0.79	0.41–1.53	0.492
1–4 times/week	1.01	0.54–1.88	0.973
Never	Ref	--	--
**Frequency of Spring Contact**			
1+/day	0.52	0.25–1.06	0.073
1–times/week	0.75	0.39–1.41	0.367
Never	Ref	--	--

^1^ COR: crude odds ratio; ^2^ CI: confidence interval; * statistically significant finding (*p* < 0.05).

**Table 7:** Multivariable mixed effects logistic regression analysis of risk factors, associated with self-reported schistosomiasis in Tanzanian schoolchildren (village included as a random effect variable).

Variable	Penalized ^1^			Reduced ^2^		
	AOR ^3^	95% CI ^4^		AOR ^3^	95% CI ^4^	
**Sex**						
Male	2.68	1.89–3.80	<0.001 *	2.69	1.90–3.80	<0.001 *
Female	Ref	--	--	Ref	--	--
**Age**	1	--	--	--	--	--
**Occupation of Parent**	1	--	--	--	--	--
**Latrine Use**						
Yes (home or neighbors)	Ref	--	--	Ref	--	--
No	2.32	0.90–5.98	0.083	2.33	0.90–6.01	0.080
**Main Source of Drinking Water**						
Safe (wells and rainwater)	Ref	--	--	Ref	--	--
Unsafe (river, dam, etc.)	3.55	2.02–6.26	<0.001 *	3.57	2.03–6.27	<0.001 *
**Wealth Index**						
Cluster 1 (poorest)	Ref	--	--	Ref	--	--
Cluster 2	0.55	0.26–1.17	0.118	0.54	0.18–1.63	0.275
Cluster 3	0.64	0.42–0.98	0.040 *	0.64	0.26–1.56	0.326
Cluster 4	0.43	0.27–0.68	<0.001 *	0.43	0.18–1.04	0.060
Cluster 5 (least poor)	0.38	0.04–3.46	0.391	0.38	0.16–0.90	0.028 *
**Frequency of Lake Contact**						
1+/day	1.65	0.37–7.38	0.513	1.66	0.59–4.67	0.341
1–4 times/week	3.25	1.80–5.89	<0.001 *	3.25	1.25–8.47	0.016 *
Never	Ref	--	--	Ref	--	--
**Frequency of River Contact**						
1+/day	0.26	0.08–0.85	0.025 *	0.26	0.12–0.56	<0.001 *
1–4 times/week	0.27	0.15–0.48	<0.001 *	0.26	0.12–0.60	0.001 *
Never	Ref	--	--	Ref	--	--
**Frequency of Spring Contact**	1	--	--	--	--	--
**Frequency of Dam Contact**	1	--	--	--	--	--
**Education of Parent**						
No formal education	0.65	0.34–1.22	0.176	0.64	0.24–1.71	0.375
Incomplete primary	0.46	0.32–0.64	<0.001 *	0.45	0.18–1.16	0.100
Primary education	0.56	0.08–4.09	0.564	0.55	0.21–1.47	0.236
Incomplete secondary	0.99	0.51–1.89	0.967	0.98	0.33–2.89	0.976
Secondary education	Ref	--	--	Ref	--	--

^1^ Penalized: Estimates are reported from a penalized (LASSO) logistic mixed effects regression model. ^2^ Reduced: Estimates are reported from an unpenalized logistic mixed effects regression model which included only factors with nonzero coefficients in the LASSO regression. ^3^ AOR: adjusted odds ratio. ^4^ CI: confidence interval; * statistically significant finding (*p* < 0.05).

## Supplementary Materials

The following are available online at www.mdpi.com/2414-6366/2/4/61/s1, Figure S1: Flow diagram of study selection process, Figure S2: Deeks funnel plot asymmetry test for publication bias for *S. haematobium* diagnostic questions: (a) blood in urine; (b) pain during urination; and (c) history of schistosomiasis, Figure S3: Deeks funnel plot asymmetry test for publication bias for *S. mansoni* diagnostic questions: (a) abdominal pain; (b) history of schistosomiasis; and (c) blood in stool; and (d) bloody diarrhea, Figure S4: Quality assessment results for diagnostic questions used in *S. haematobium* meta-analysis: (a) blood in urine; (b) pain during urination; and (c) history of schistosomiasis, Figure S5: Quality assessment results for diagnostic questions used in *S. mansoni* meta-analysis: (a) blood in stool; (b) bloody diarrhea; (c) history of schistosomiasis; and (d) abdominal pain, Figure S6: Sensitivity forest plot, specificity forest plot, and SROC plot for blood in urine question (*S. haematobium*): (a) Sensitivity forest plot; (b) specificity forest plot; and (c) SROC curve with summary sensitivity and false positive rate (1-specificity) (circle) and the 95% confidence region (ellipse). Each triangle represents the summary sensitivity and false positive rate from one study, Figure S7: Sensitivity forest plot, specificity forest plot, and SROC plot for history of schistosomiasis question (*S. haematobium*): (a) Sensitivity forest plot; (b) specificity forest plot; and (c) SROC curve with summary sensitivity and false positive rate (1-specificity) (circle) and the 95% confidence region (ellipse). Each triangle represents the summary sensitivity and false positive rate from one study, Figure S8: Sensitivity forest plot, specificity forest plot, and SROC plot for blood in stool question (*S. mansoni*): (a) Sensitivity forest plot; (b) specificity forest plot; and (c) SROC curve with summary sensitivity and false positive rate (1-specificity) (circle) and the 95% confidence region (ellipse). Each triangle represents the summary sensitivity and false positive rate from one study, Figure S9: Sensitivity forest plot, specificity forest plot, and SROC plot for bloody diarrhea question (*S. mansoni*): (a) Sensitivity forest plot; (b) specificity forest plot; and (c) SROC curve with summary sensitivity and false positive rate (1-specificity) (circle) and the 95% confidence region (ellipse). Each triangle represents the summary sensitivity and false positive rate from one study, Figure S10: Sensitivity forest plot, specificity forest plot, and SROC plot for abdominal pain question (*S. mansoni*): (a) Sensitivity forest plot; (b) specificity forest plot; and (c) SROC curve with summary sensitivity and false positive rate (1-specificity) (circle) and the 95% confidence region (ellipse). Each triangle represents the summary sensitivity and false positive rate from one study, Figure S11: Sensitivity forest plot, specificity forest plot, and SROC plot for history of schistosomiasis (*S. mansoni*): (a) Sensitivity forest plot; (b) specificity forest plot; and (c) SROC curve with summary sensitivity and false positive rate (1-specificity) (circle) and the 95% confidence region (ellipse). Each triangle represents the summary sensitivity and false positive rate from one study, Figure S12: Proportion of students classified as *S. haematobium-* (red) or *S. mansoni-* (blue) positive in this study using different LR+ threshold values. The proportion classified as infected is shown to decrease as the LR+ threshold value is raised because of reductions in false positives. An LR+ of 3.5 was selected as the threshold value in this study to balance the need for obtaining adequate numbers of positive cases while keeping the false positive rate sufficiently low, Figure S13: Distribution of individual wealth scores among 1704 Tanzanian schoolchildren with schistosomiasis status indicated by color: positive (blue) and negative (red), Table S1: Characteristics of studies with data for the diagnostic questions used in the *S. haematobium* meta-analyses, Table S2: Characteristics of studies with data for the diagnostic questions used in the *S. mansoni* meta-analyses, Table S3: Average asset ownership by cluster in the constructed wealth index.
