# Supplementary material for: Biosocial Determinants of Persistent Schistosomiasis among Schoolchildren in Tanzania despite Repeated Treatment
Source: Trop Med Infect Dis. 2017 Dec 4;2(4):61. doi: 10.3390/tropicalmed2040061 (PMC6082061; doi:10.3390/tropicalmed2040061)
Supplement: Supplementary file 1 [file tropicalmed-02-00061-s001.pdf]

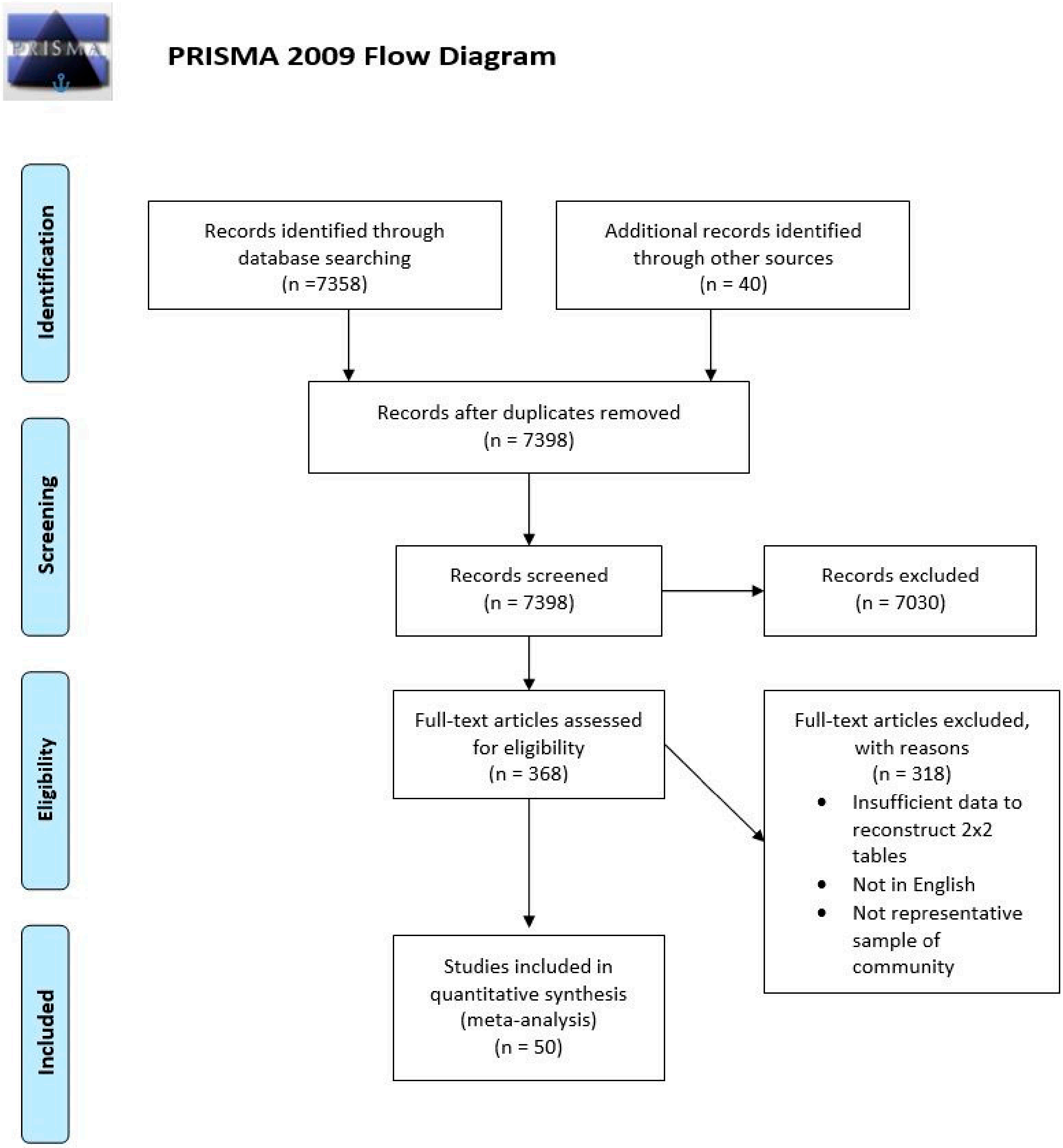

Figure S1. Flow diagram of study selection process

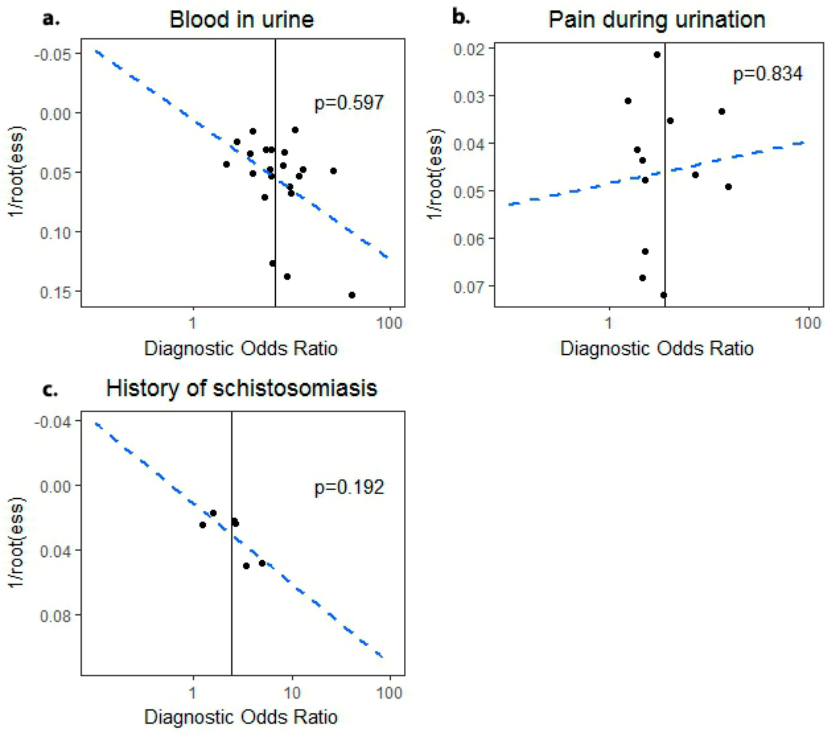

Figure S2. Deeks funnel plot asymmetry test for publication bias for *S. haematobium* diagnostic questions: **(a)** blood in urine; **(b)** pain during urination; and **(c)** history of schistosomiasis.

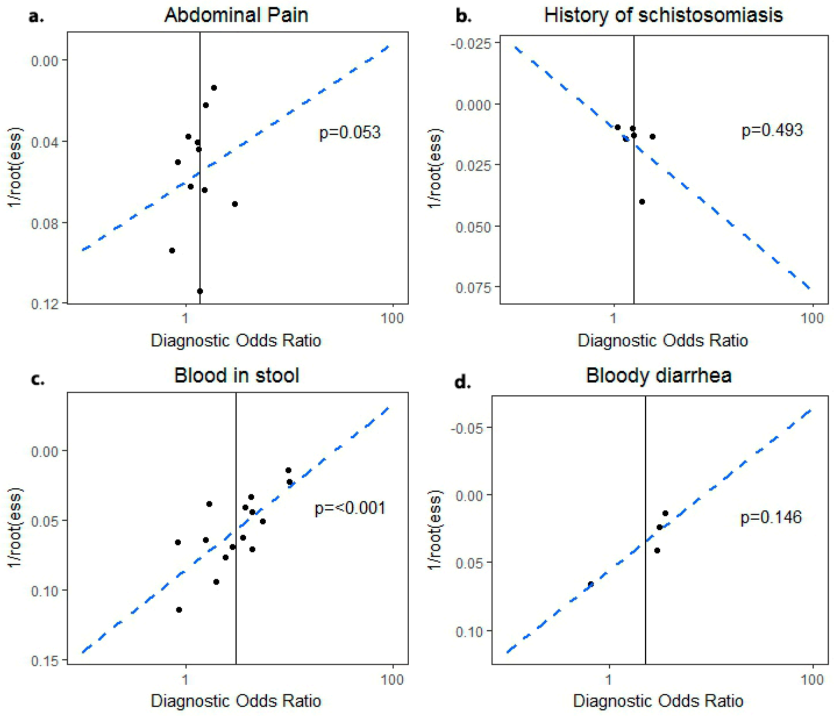

Figure S3. Deeks funnel plot asymmetry test for publication bias for *S. mansoni* diagnostic questions: **(a)** abdominal pain; **(b)** history of schistosomiasis; and **(c)** blood in stool; and **(d)** bloody diarrhea.

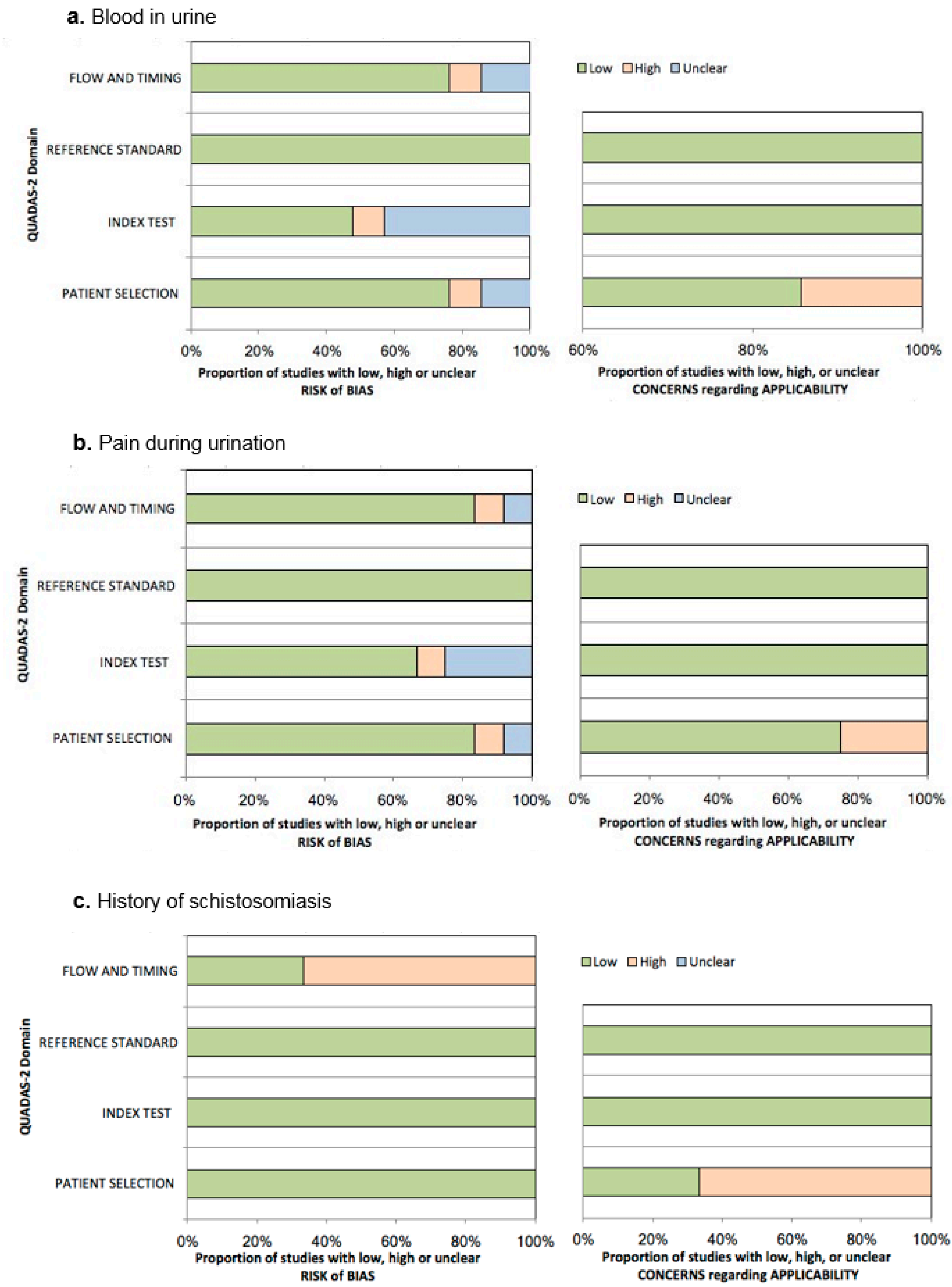

Figure S4. Quality assessment results for diagnostic questions used in *S. haematobium* meta-analysis: **(a)** blood in urine; **(b)** pain during urination; and **(c)** history of schistosomiasis.

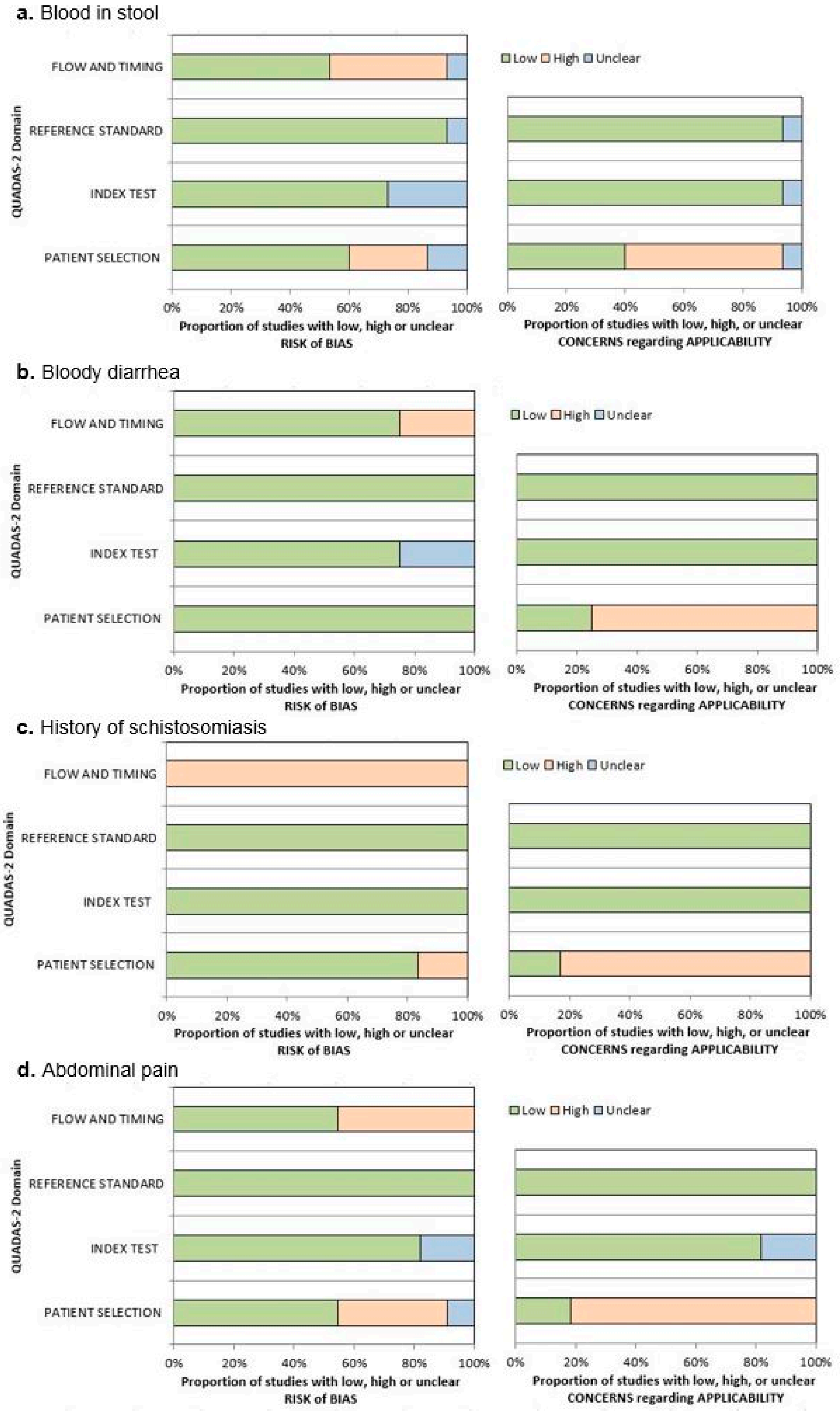

Figure S5. Quality assessment results for diagnostic questions used in *S. mansoni* meta-analysis: **(a)** blood in stool; **(b)** bloody diarrhea; **(c)** history of schistosomiasis; and **(d)** abdominal pain.

a.

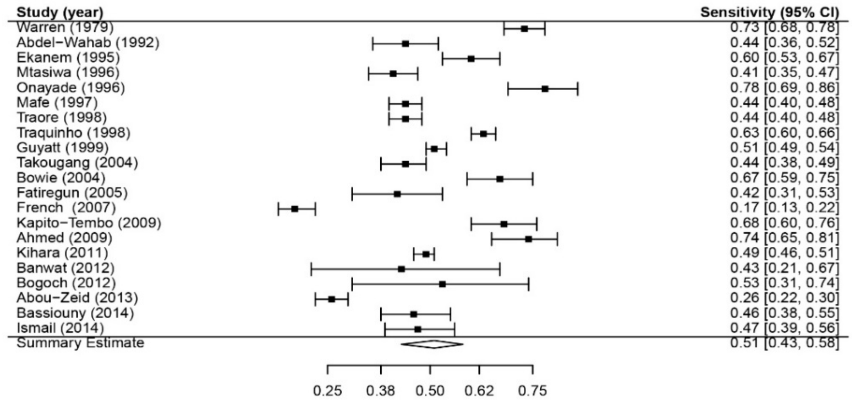

b.

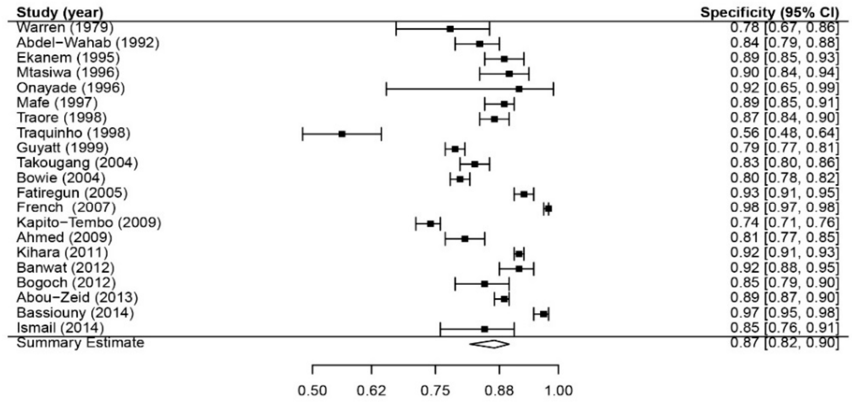

c.

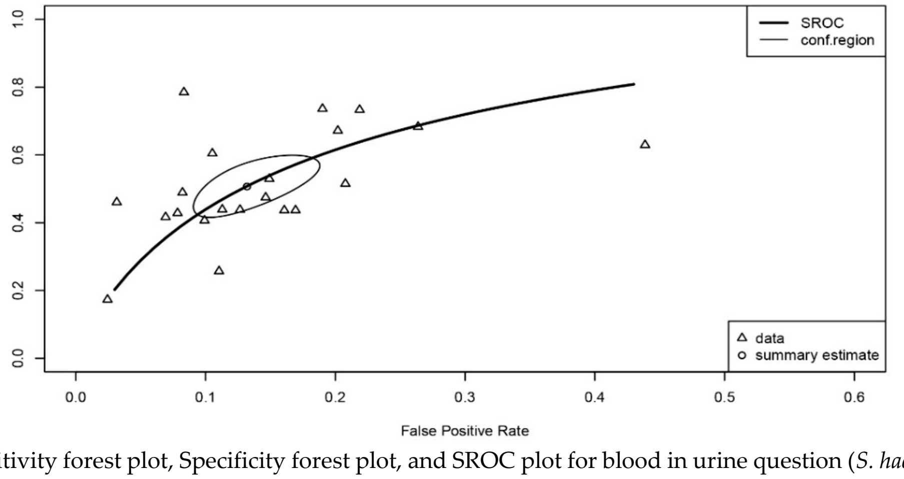

Figure S6. Sensitivity forest plot, Specificity forest plot, and SROC plot for blood in urine question (*S. haematobium*): **(a)** Sensitivity forest plot; **(b)** Specificity forest plot; and **(c)** SROC curve with summary sensitivity and false positive rate (1-specificity) (circle) and the 95% confidence region (ellipse). Each triangle represents the summary sensitivity and false positive rate from one study.

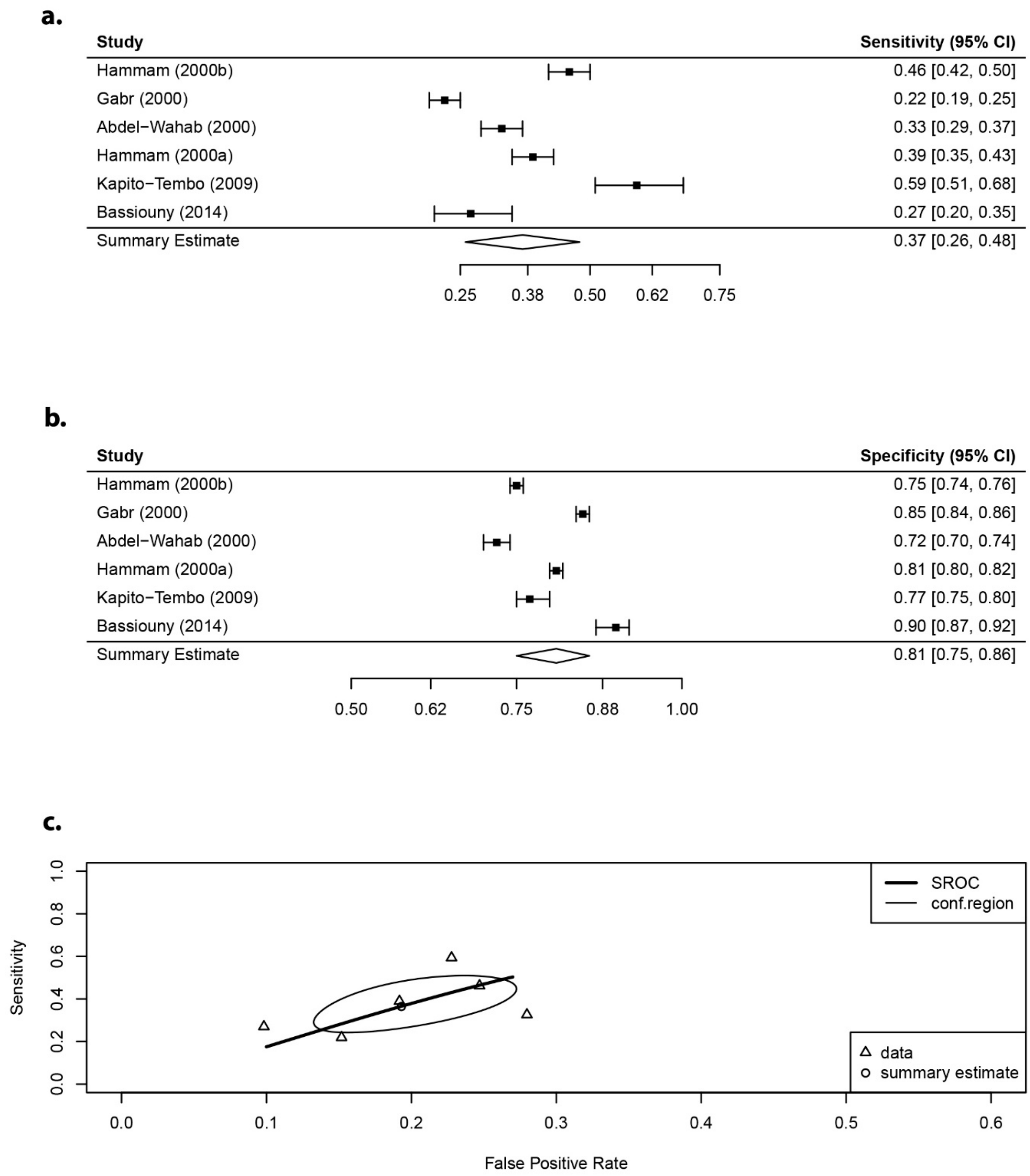

Figure S7. Sensitivity forest plot, Specificity forest plot, and SROC plot for history of schistosomiasis question (*S. haematobium*): **(a)** Sensitivity forest plot; **(b)** Specificity forest plot; and **(c)** SROC curve with summary sensitivity and false positive rate (1-specificity) (circle) and the 95% confidence region (ellipse). Each triangle represents the summary sensitivity and false positive rate from one study.

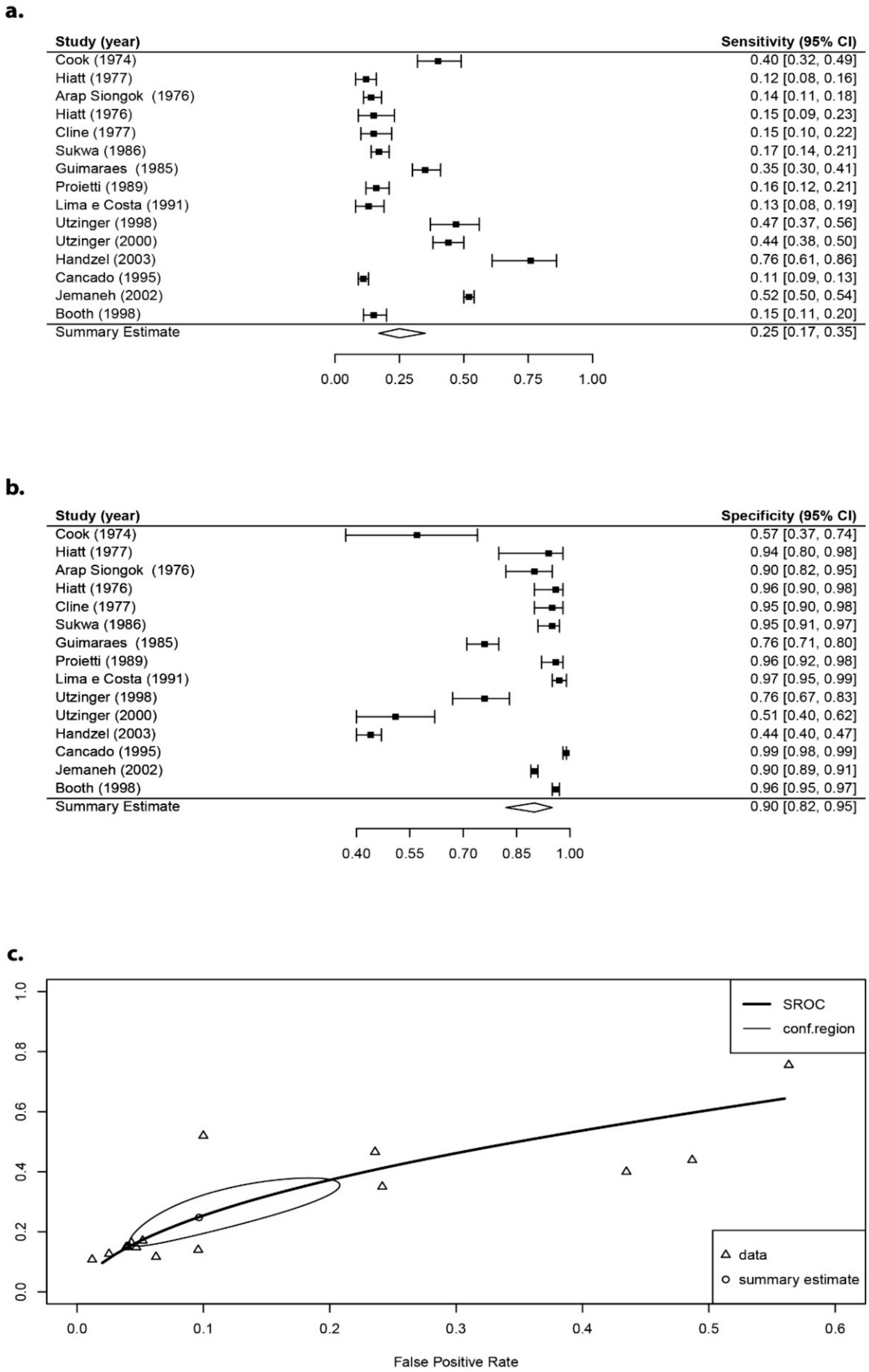

Figure S8. Sensitivity forest plot, Specificity forest plot, and SROC plot for blood in stool question (*S. mansoni*): **(a)** Sensitivity forest plot; **(b)** Specificity forest plot; and **(c)** SROC curve with summary sensitivity and false positive rate (1-specificity) (circle) and the 95% confidence region (ellipse). Each triangle represents the summary sensitivity and false positive rate from one study.

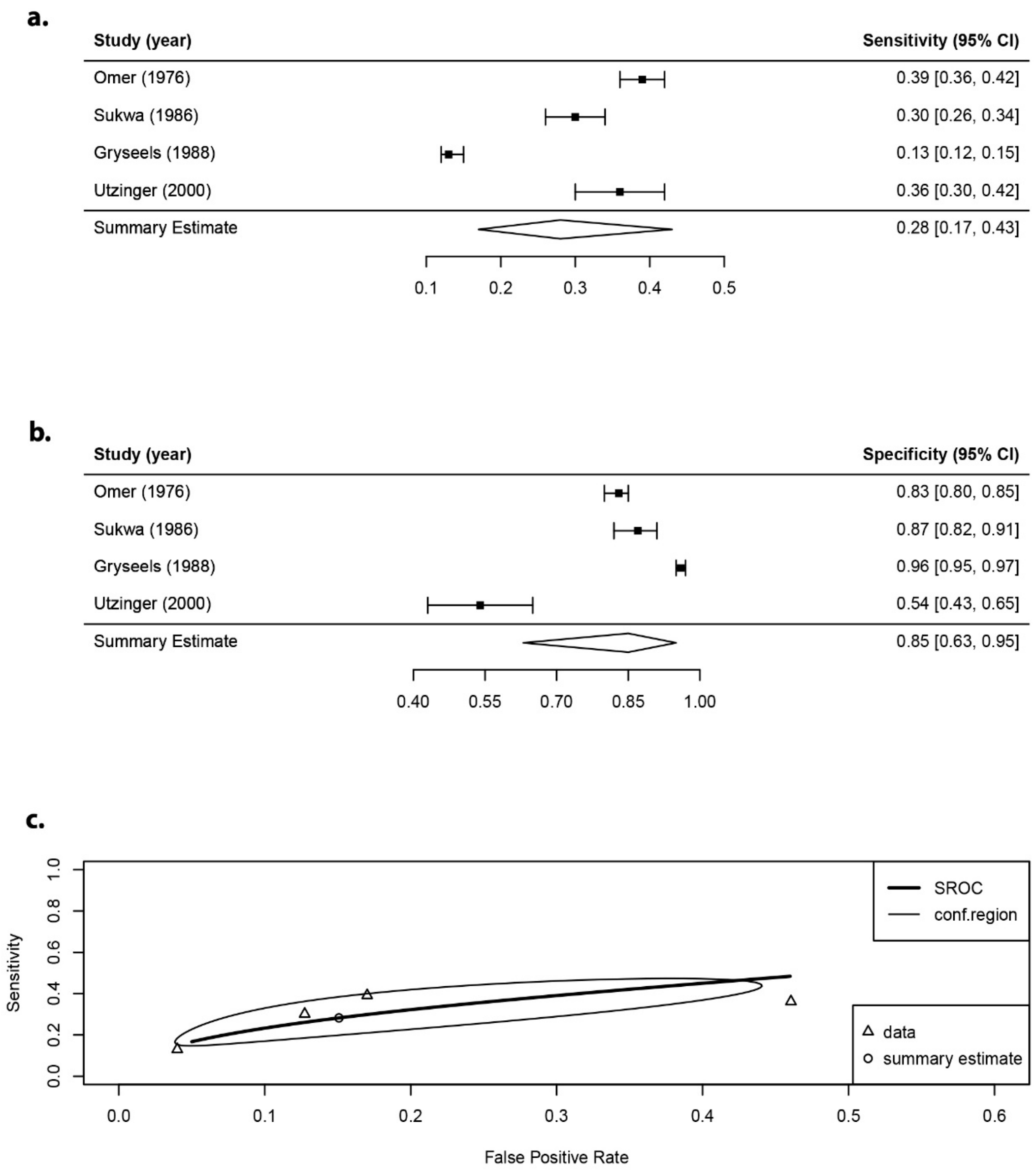

Figure S9. Sensitivity forest plot, Specificity forest plot, and SROC plot for bloody diarrhea question (*S. mansoni*): **(a)** Sensitivity forest plot; **(b)** Specificity forest plot; and **(c)** SROC curve with summary sensitivity and false positive rate (1-specificity) (circle) and the 95% confidence region (ellipse). Each triangle represents the summary sensitivity and false positive rate from one study.

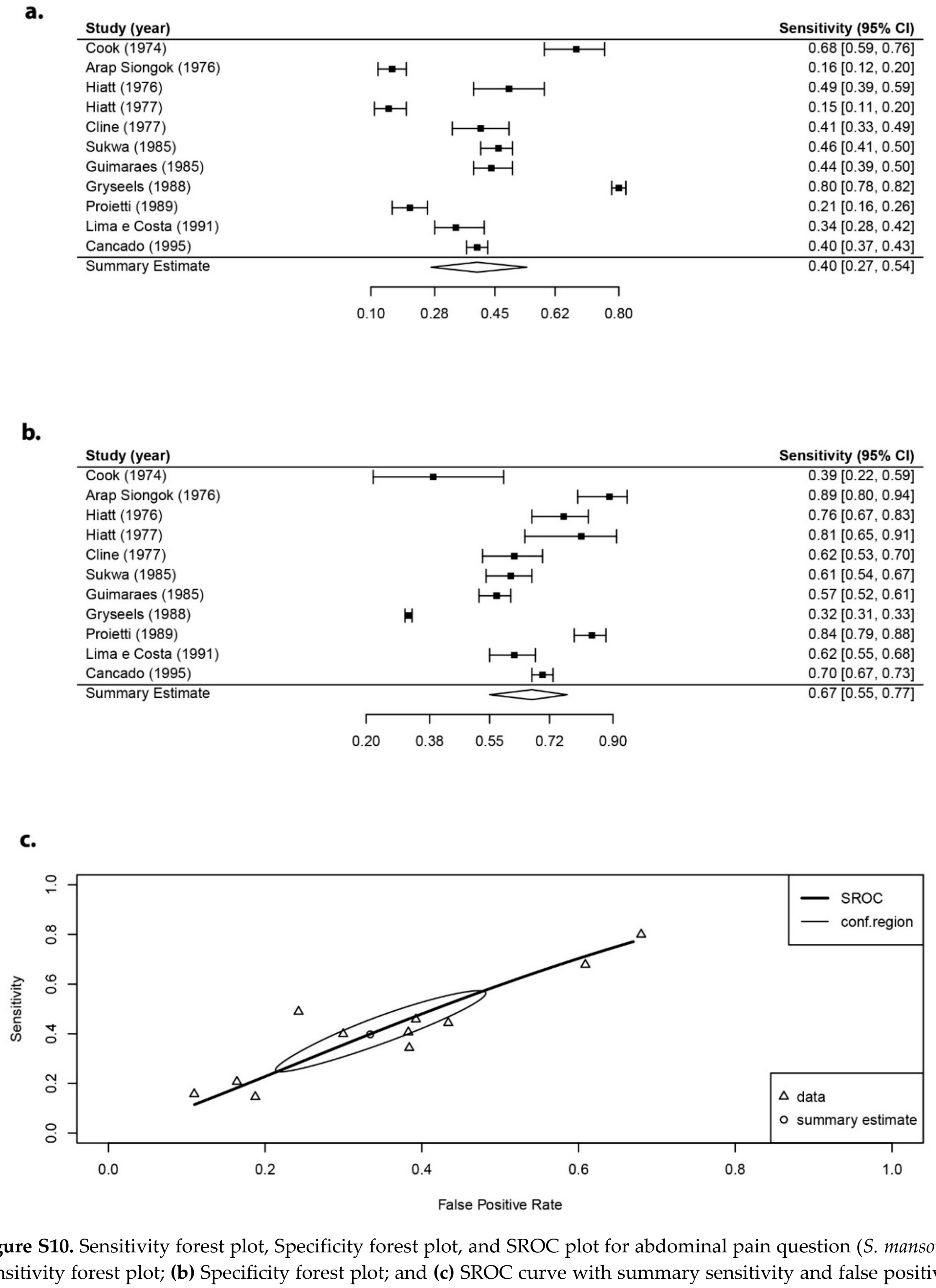

Figure S10. Sensitivity forest plot, Specificity forest plot, and SROC plot for abdominal pain question (*S. mansoni*): **(a)** Sensitivity forest plot; **(b)** Specificity forest plot; and **(c)** SROC curve with summary sensitivity and false positive rate (1-specificity) (circle) and the 95% confidence region (ellipse). Each triangle represents the summary sensitivity and false positive rate from one study.

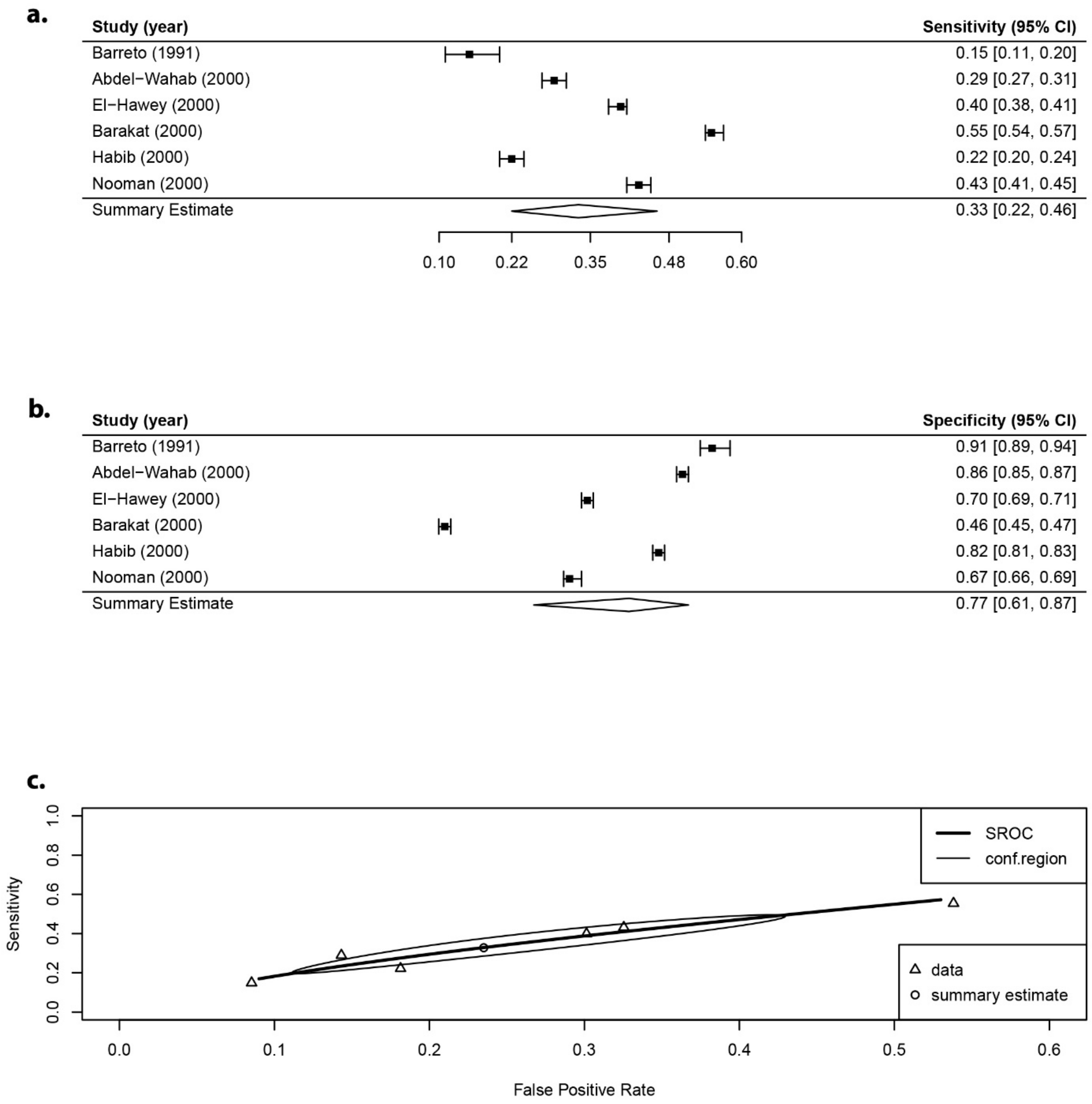

Figure S11. Sensitivity forest plot, Specificity forest plot, and SROC plot for history of schistosomiasis (*S. mansoni*). **(a)** Sensitivity forest plot; **(b)** Specificity forest plot; and **(c)** SROC curve with summary sensitivity and false positive rate (1-specificity) (circle) and the 95% confidence region (ellipse). Each triangle represents the summary sensitivity and false positive rate from one study.

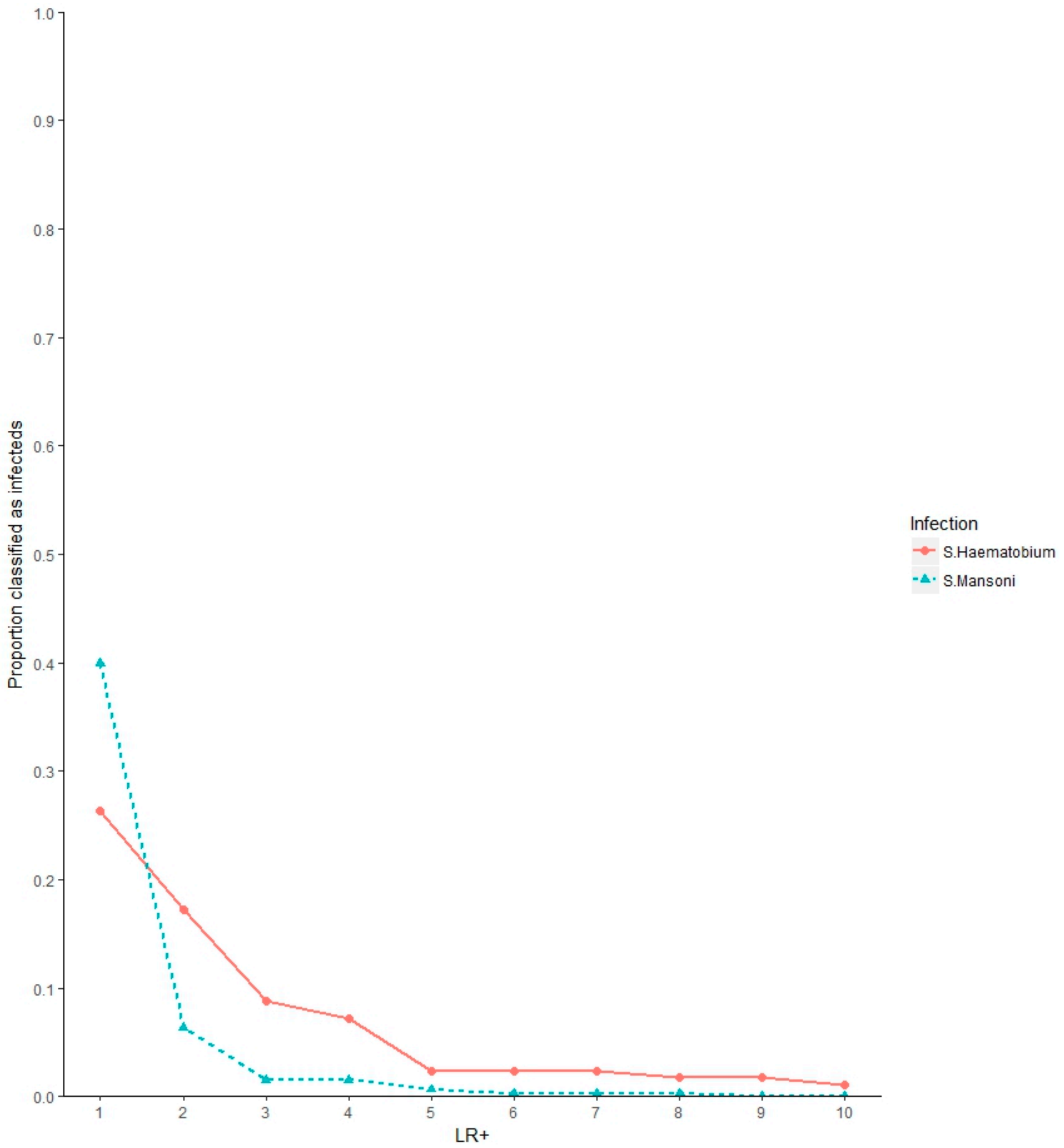

Figure S12. Proportion of students classified as *S. haematobium-* (red) or *S. mansoni-* (blue) positive in this study using different LR+ threshold values. The proportion classified as infected is shown to decrease as the LR+ threshold value is raised because of reductions in false positives. A LR+ of 3.5 was selected as the threshold value in this study to balance the need for obtaining adequate numbers of positive cases while keeping the false positive rate sufficiently low.

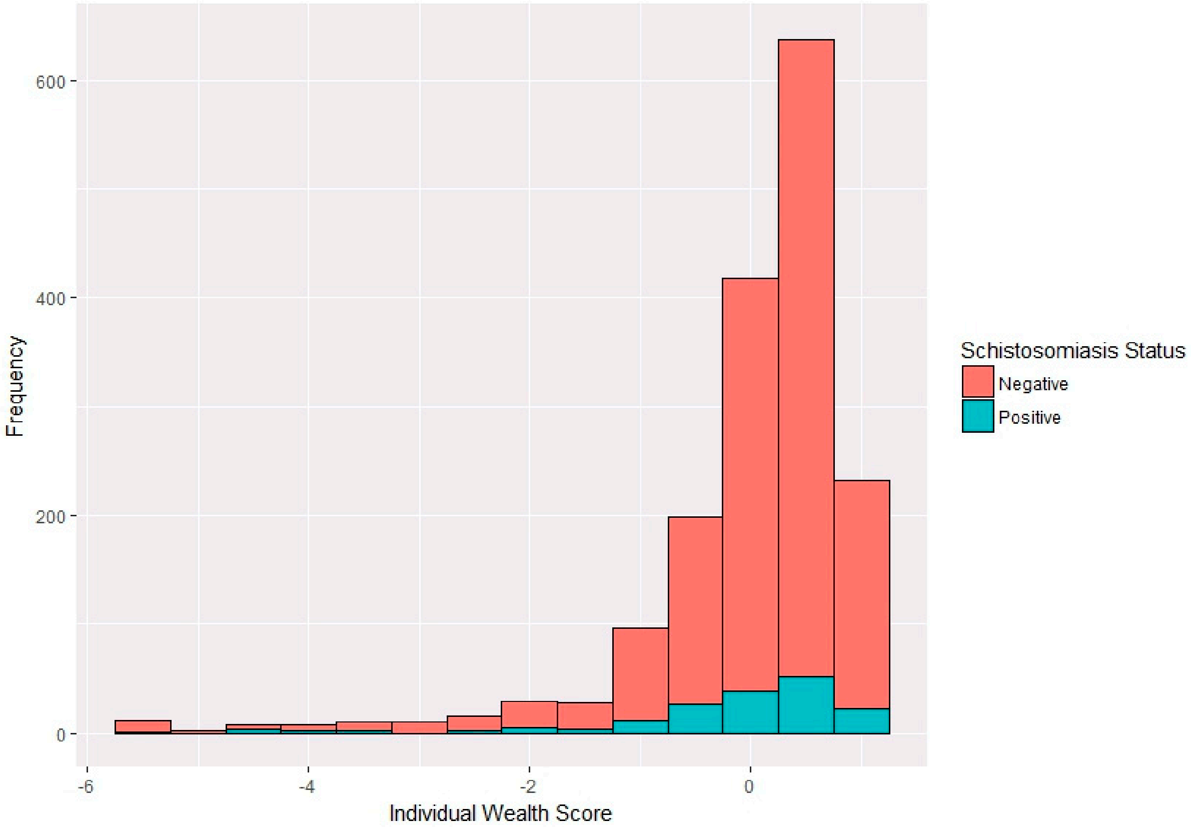

Figure S13. Distribution of individual wealth scores among 1704 Tanzanian schoolchildren with schistosomiasis status indicated by color: positive (blue) and negative (red).

Table S1. Characteristics of studies with data for the diagnostic questions used in the *S. haematobium* meta-analyses

Diagnostic Question	First Author	Year of Publication	Country	Sample size	Prevalence	Ages	TP	FP	FN	TN
Blood in urine	Warren	1979	Kenya	390	83.6%	Schoolchildren	239	14	87	50
Blood in urine	Abdel-Wahab	1992	Egypt	422	33.6%	Schoolchildren	62	45	80	235
Blood in urine	Ekanem	1995	Nigeria	462	38.3%	Schoolchildren	107	30	70	255
Blood in urine	Mtasiwa	1996	Tanzania	404	67.6%	Schoolchildren	111	13	162	118
Blood in urine	Onayade	1996	Nigeria	105	88.6%	Schoolchildren	73	1	20	11
Blood in urine	Mafe	1997	Nigeria	1024	57.6%	All	259	49	331	385
Blood in urine	Traore	1998	Mali	1041	55.2%	All	252	59	323	407
Blood in urine	Traquinho	1998	Mozambique	994	84.4%	Schoolchildren	528	68	311	87
Blood in urine	Guyatt	1999	Tanzania	3928	58.1%	Schoolchildren	1175	342	1107	1304
Blood in urine	Takougang	2004	Cameroon	871	36.3%	Schoolchildren	138	94	178	461
Blood in urine	Bowie	2004	Malawi	1565	8.6%	Schoolchildren	90	289	44	1142
Blood in urine	Fatiregun	2005	Nigeria	592	12.2%	Schoolchildren	30	36	42	484
Blood in urine	French	2007	Tanzania	1976	13.2%	Schoolchildren	45	42	215	1674
Blood in urine	Kapito-Tembo	2009	Malawi	1139	10.8%	Schoolchildren	84	268	39	748
Blood in urine	Ahmed	2009	Yemen	515	21.4%	Schoolchildren	81	77	29	328
Blood in urine	Kihara	2011	Kenya	6183	24.5%	Schoolchildren	741	384	774	4284
Blood in urine	Banwat	2012	Nigeria	218	6.4%	Schoolchildren	6	16	8	188
Blood in urine	Bogoch	2012	Ghana	198	8.6%	All	9	27	8	154
Blood in urine	Abou-Zeid	2013	Sudan	2302	23.7%	Schoolchildren	140	194	405	1563
Blood in urine	Bassiouny	2014	Yemen	696	18.1%	Schoolchildren	58	18	68	552
Blood in urine	Ismail	2014	Sudan	200	59.0%	Schoolchildren	56	12	62	70
Pain during urination	Warren	1979	Kenya	390	83.6%	Schoolchildren	189	25	137	39
Pain during urination	Pugh	1980	Nigeria	4296	15.2%	All	171	380	484	3261
Pain during urination	King	1988	Kenya	639	64.8%	All	177	63	237	162
Pain during urination	Ekanem	1995	Nigeria	510	34.7%	Schoolchildren	90	41	87	292
Pain during urination	Traquinho	1998	Mozambique	994	84.4%	Schoolchildren	456	55	383	100
Pain during urination	Traore	1998	Mali	1041	55.2%	All	223	134	352	332
Pain during urination	Takougang	2004	Cameroon	871	36.3%	Schoolchildren	142	91	174	464
Pain during urination	Fatiregun	2005	Nigeria	592	12.2%	Schoolchildren	18	65	54	455
Pain during urination	French	2007	Tanzania	1976	13.2%	Schoolchildren	43	25	217	1691
Pain during urination	Kapito-Tembo	2009	Malawi	1124	10.9%	Schoolchildren	46	204	77	797
Pain during urination	Bassiouny	2014	Yemen	696	18.1%	Schoolchildren	99	108	27	462
Pain during urination	Ismail	2014	Sudan	200	59.0%	Schoolchildren	82	32	36	50
History of schistosomiasis	Hammam	2000	Egypt	10419	5.4%	All	262	2434	305	7418
History of schistosomiasis	Gabr	2000	Egypt	10331	9.3%	All	210	1423	749	7949
History of schistosomiasis	Abdel-Wahab	2000	Egypt	3470	14.5%	All	164	830	339	2137
History of schistosomiasis	Hammam	2000	Egypt	7665	6.5%	All	195	1374	306	5790
History of schistosomiasis	Kapito-Tembo	2009	Malawi	1133	10.9%	Schoolchildren	73	230	50	780
History of schistosomiasis	Bassiouny	2014	Yemen	696	18.1%	Schoolchildren	34	56	92	514

Table S2. Characteristics of studies with data for the diagnostic questions used in the *S. mansoni* meta-analyses.

Diagnostic Question	First Author	Year of Publication	Country	Sample size	Prevalence	Ages	TP	FP	FN	TN
Abdominal pain	Cook	1974	St Lucia	138	83.3%	Schoolchildren	78	14	37	9
Abdominal pain	Arap Siongok	1976	Kenya	416	82.5%	All	54	8	289	65
Abdominal pain	Hiatt	1976	Ethiopia	197	47.7%	All	46	25	48	78
Abdominal pain	Hiatt	1977	Ethiopia	272	88.2%	Schoolchildren	35	6	205	26
Abdominal pain	Cline	1977	Puerto Rico	256	50.0%	All	52	49	76	79
Abdominal pain	Sukwa	1985	Zambia	703	69.6%	All	224	84	265	130
Abdominal pain	Guimaraes	1985	Brazil	696	44.7%	All	138	167	173	218
Abdominal pain	Gryseels	1988	Burundi	6203	32.8%	All	1628	2834	407	1334
Abdominal pain	Proietti	1989	Brazil	512	50.0%	All	53	42	203	214
Abdominal pain	Lima e Costa	1991	Brazil	403	41.2%	All	57	91	109	146
Abdominal pain	Cancado	1995	Brazil	1971	53.3%	All	419	276	631	645
Bloody diarrhea	Omer	1976	Sudan	1748	48.2%	All	330	154	513	751
Bloody diarrhea	Sukwa	1986	Zambia	693	69.4%	All	145	27	336	185
Bloody diarrhea	Gryseels	1988	Burundi	6203	32.8%	All	265	167	1770	4001
Bloody diarrhea	Utzinger	2000	Cote d'Ivoire	322	76.4%	Schoolchildren	89	35	157	41
Blood in stool	Cook	1974	St Lucia	138	83.3%	Schoolchildren	46	10	69	13
Blood in stool	Hiatt	1977	Ethiopia	272	88.2%	Schoolchildren	28	2	212	30
Blood in stool	Arap Siongok	1976	Kenya	416	82.5%	All	48	7	295	66
Blood in stool	Hiatt	1976	Ethiopia	197	47.7%	All	14	4	80	99
Blood in stool	Cline	1977	Puerto Rico	256	50.0%	All	19	6	109	122
Blood in stool	Sukwa	1986	Zambia	693	69.4%	All	82	11	399	201
Blood in stool	Guimaraes	1985	Brazil	696	44.7%	All	109	93	202	292
Blood in stool	Proietti	1989	Brazil	512	50.0%	All	42	11	214	245
Blood in stool	Lima e Costa	1991	Brazil	403	41.2%	All	21	6	145	231
Blood in stool	Utzinger	1998	Cote d'Ivoire	209	49.3%	Schoolchildren	48	25	55	81
Blood in stool	Utzinger	2000	Cote d'Ivoire	322	76.4%	Schoolchildren	108	37	138	39
Blood in stool	Handzel	2003	Kenya	748	6.0%	Schoolchildren	34	396	11	307
Blood in stool	Cancado	1995	Brazil	1971	53.3%	Community	113	11	937	910
Blood in stool	Jemaneh	2002	Ethiopia	8006	20.9%	Unknown	870	633	803	5700
Blood in stool	Booth	1998	Tanzania	4130	5.8%	Unknown	36	156	204	3734
History of schistosomiasis	Barreto	1991	Brazil	778	27.6%	Schoolchildren	32	48	183	515
History of schistosomiasis	Abdel-Wahab	2000	Egypt	6901	28.0%	All	560	711	1375	4255
History of schistosomiasis	El-Hawey	2000	Egypt	9661	41.9%	All	1616	1691	2434	3920
History of schistosomiasis	Barakat	2000	Egypt	11272	40.4%	All	2524	3617	2027	3104
History of schistosomiasis	Habib	2000	Egypt	7085	20.9%	All	329	1016	1154	4586
History of schistosomiasis	Nooman	2000	Egypt	6246	42.0%	All	1133	1178	1493	2442

Table S3. Average asset ownership by cluster in the constructed wealth index.

Asset	I (Poorest)	II	III	IV	V (Least poor)
House	7.9%	46.6%	95.4%	100.0%	100.0%
Latrine	7.9%	71.2%	98.0%	100.0%	100.0%
Land	31.6%	53.4%	77.2%	87.1%	100.0%
Radio	34.2%	37.0%	46.0%	58.7%	98.7%
TV	13.1%	26.0%	13.9%	15.4%	16.4%
Motorcycle	0.0%	15.1%	18.5%	25.7%	30.3%
Bicycle	23.7%	53.4%	30.4%	63.0%	99.8%
Phone	39.5%	52.1%	42.7%	87.1%	100.0%
Fridge	5.3%	15.1%	9.3%	8.2%	10.4%
